# Two meta-analyses of the association between atopic diseases and core symptoms of attention deficit hyperactivity disorder

**DOI:** 10.1038/s41598-022-07232-1

**Published:** 2022-03-01

**Authors:** Yu-Chieh Chuang, Ching-Yun Wang, Wei-Lieh Huang, Liang-Jen Wang, Ho-Chang Kuo, Yang-Ching Chen, Yu-Jui Huang

**Affiliations:** 1 Department of General Medicine, Shuang Ho Hospital, Taipei Medical University, New Taipei City, Taiwan; 2 Department of Pediatrics, Taipei Medical University Hospital, Taipei, Taiwan; 3 Department of Psychiatry, National Taiwan University Hospital Yunlin Branch, Yunlin, Taiwan; 4 Department of Psychiatry, National Taiwan University Hospital, Taipei, Taiwan; 5 Department of Psychiatry, College of Medicine, National Taiwan University, Taipei, Taiwan; 6 Graduate Institute of Clinical Medicine, College of Medicine, National Taiwan University, Taipei, Taiwan; 7 Department of Child and Adolescent Psychiatry, Kaohsiung Chang Gung Memorial Hospital and Chang Gung University College of Medicine, Kaohsiung, Taiwan; 8 School of Medicine, College of Medicine, Chang Gung University, Taoyuan, Taiwan; 9 Department of Pediatrics, Kaohsiung Chang Gung Memorial Hospital and Chang Gung University College of Medicine, Kaohsiung, Taiwan; 10 Kawasaki Disease Center, Kaohsiung Chang Gung Memorial Hospital, Kaohsiung, Taiwan; 11 Department of Family Medicine, Taipei Medical University Hospital, Taipei, Taiwan; 12 Department of Family Medicine, School of Medicine, College of Medicine, Taipei Medical University, Taipei, Taiwan; 13 Department of Psychiatry and Psychiatric Research Center, Taipei Medical University Hospital, Taipei, Taiwan

**Keywords:** Immunology, Psychology, Medical research, Signs and symptoms

## Abstract

Studies in the field of neuroscience and psychology have hypothesized that a causal association exists between atopic diseases and attention-deficit/hyperactivity disorder (ADHD). Previous systematic reviews and meta-analyses have reported a higher risk of ADHD in children with atopic diseases; however, the relationship between ADHD symptoms and atopic diseases remains unclear. We systematically reviewed observational cross-sectional and longitudinal studies to investigate the relationship between atopic diseases and ADHD symptom severity (hyperactivity/impulsivity and inattention). The majority of studies showed a statistically significant association between atopic diseases and both ADHD symptoms, with substantial heterogeneity in the outcome of hyperactivity/impulsivity. Remarkably decreased heterogeneity and statistical significance were observed in the second meta-analysis of ADHD-related behavior symptoms in atopic patients without ADHD. Our study indicated that atopic diseases not only associated with ADHD but also ADHD symptoms severity. This association was even observed in children with subthreshold ADHD, indicating that atopic diseases may play a role in the spectrum of ADHD symptom severity. **Trial registration**: This study was registered on PROSPERO (registration ID: CRD42020213219).

## Introduction

In the past decade, atopic diseases have been a cause of increased concern because of their substantially high prevalence (approximately 20 to 40%) worldwide^1^, with more prominent increasing trend in developing countries^2^. Atopic march, defined as the development of atopic dermatitis in infancy and subsequent allergic rhinitis and asthma in later childhood, can cause fatigue; attention, learning, and memory deficits; and depression. Moreover, atopic diseases have been reported to considerably affect children’s sleep, school performance, development, and quality of life^3,4^. In addition, recent studies have reported that the parents or main caregivers of children with atopic diseases, particularly those with multiple atopic diseases, experience considerable stress and psychosocial burden^4–8^ Thus, increasing public attention has been focused on the management of atopic diseases^8^.

Attention-deficit/hyperactivity disorder (ADHD) is among the most common neuropsychiatric disorders in children and adolescents, with a prevalence of approximately 5%. ADHD is characterized by hyperactivity, impulsivity, and inattention that lead to not only functional impairment but also executive function impairment and emotional dysregulation^9,10^. ADHD can be caused by multiple factors (e.g., genetic, epigenetic, and environmental). Moreover, studies have indicated that ADHD occurs due to immature brain development, and it may also be accompanied by other neurobehavioral developmental delays and even intellectual disability^10,11^. Many studies have reported that ADHD symptoms were still observed in adulthood in a majority of patients with ADHD, even in those with adult ADHD who did not meet the criteria for ADHD in their childhood^12–14^. Adult patients with ADHD experience a financial burden and may even require considerable support^14^. These findings indicate that ADHD is a matter of considerable concern in child and adolescent psychiatry.

A previous study investigated the relationship between atopic diseases and ADHD^15^. A higher risk of ADHD was noted in patients with all three atopic diseases, namely atopic dermatitis, allergic rhinitis, and asthma (i.e., atopic march)^16,17^. In addition, children with both atopic diseases and ADHD had an increased risk of developing more severe ADHD symptoms compared with those with only ADHD^18^. Also, the association between atopic diseases and ADHD-related behavioral symptoms^19^, which some researches mentioned as subthreshold ADHD^20^, has been investigated recently. Although several meta-analyses and systematic reviews have strongly supported the relationship between atopic diseases and ADHD^17,21,22^, they still had many limitations. The heterogeneity observed in some systematic reviews might have affected their findings; for instance, some reviews did not include studies with standard inclusion and exclusion criteria, and whether the atopic and unexposed groups included patients diagnosed as having ADHD remained unclear^16,21^. In addition, few studies have reported a relationship between atopic diseases and subthreshold ADHD. Moreover, no systematic review and meta-analysis has examined whether atopic diseases affect the severity of both hyperactivity/impulsivity and inattention—the two main symptoms and diagnostic criteria related to ADHD.

In this systematic review and meta-analysis, we reviewed studies examining the relationship between atopic diseases and the severity of ADHD symptoms including hyperactivity/impulsivity and inattention. In addition, we explored more precise value of this relationship by defining inclusion and exclusion criteria for groups. By doing so, we could exclude some confounding effects and provide additional information regarding the relationship between atopic diseases and subthreshold ADHD; this involved examining the data of children without a previous diagnosis of ADHD but who had an association of developing more severe ADHD symptoms.

## Results

### Search results

Through our literature search, we identified 4722 studies (2872, 1504, 345, and 1 from PubMed + Medline, Embase, Psycinfo, and register, respectively). Fifteen additional studies were identified through other sources. Using EndNote, we found that 671 studies were duplicates and we thus excluded them. Subsequently, we screened the titles and abstract for eligibility of the remaining 4066 studies. During title and abstract screening, we removed 630 because they did not include a relevant unexposed group and were letters or replies. Additionally, we excluded 1077 studies because they were not original research. Moreover, 1222 studies were excluded because they were not relevant to our research. Subsequently, after reviewing the full texts of the remaining 1136 studies, we excluded 1098 studies, 78 of which had missing data or full texts, 811 had nonrelevance, and 210 were not case–control, cross-sectional, or cohort studies. Finally, we included the remaining 38 studies in this systematic review for qualitative synthesis, and 16 of them were included in the quantitative review and meta-analysis. The procedure for study inclusion is shown in Appendix B in the Supplementary Materials.

### Qualitative systematic review

In 38 studies including 117,181,049 participants in our qualitative systemic review, the researchers examined the relationship between atopic diseases and ADHD symptoms severity by displaying prevalence of ADHD in patients with atopy, odds ratios (OR) of Attention deficit disorder (ADD)/ADHD in children with atopy, or calculating scores for hyperactivity/impulsivity, inattention, or total ADHD symptoms. Table 1 lists the characteristics of included studies. Most of the studies assessed atopic diseases by using parental questionnaires or based on their diagnosis by physicians or dermatologists according to specific diagnostic criteria, including the Global Initiative for Asthma guidelines, Allergic Rhinitis and its Impact on Asthma guidelines, and UK Working Party criteria. Seven study groups conducted an advanced additional test for atopy such as the skin-prick test and blood sampling for serum-specific IgE (MAST or Phadiatop tests). The included studies used different outcome scales including the Conners’ Parent Rating Scale; ADHD Rating Scale; Swanson, Nolan, and Pelham IV Scale; Child Behavior Checklist, Early Childhood Inventory-4, the German ADHD Rating scale (FBB-HKS), and the Strengths and Difficulties Questionnaire (SDQ). Despite the variability in outcome scales, most scales were designed on the basis of the DSM-IV criteria. Eight studies adopted the SDQ, which is not derived from the DSM-IV criteria; however, the SDQ has been reported to be relevant to the DSM-IV^23–25^. Most of the studies investigated the effect of confounding factors, including age, sex, multiple atopic diseases, the comorbidity with ADHD, severity of atopic diseases, persistent atopic diseases, and stressful parenting. Nineteen of the twenty studies examining the outcome of total ADHD symptoms reported a significant relationship between the severity of ADHD symptoms and atopic diseases. Furthermore, 9 of the 15 studies examining the outcome of hyperactivity and 13 of the 16 studies examining the outcome of inattention demonstrated a significant relationship between the severity of the examined ADHD symptoms and atopic diseases.

**Table 1:** Studies examining the relationship between atopic diseases and hyperactivity in the qualitative analysis.

Study name	Outcome (95% CI)	Study design	Country/City	Ethnicity	Number of participants/Exposed group/Unexposed group	Age (mean of the exposed group)/sex (male, %)	Diagnostic criteria for ADHD	Atopic disease/method used for assessing atopic diseases	Adjustment for confounding factors
Yuksel et al. 2008^58^	Continuous data for HI and IN*	Cross-sectional	Manisa, Turkey	Not reported	100/62/38	7–12 years (9.2)/57%	DSM-IV	Asthma/Physician’s diagnosis (GINA 2006)	No
Camfferman et al. 2010^26^	Continuous data for HI* and total ADHD symptoms*	Cross-sectional	South Australia, Australia	Not reported	107/77/30	6–16 years (9.9)/46.72%	Conner’s Parent Rating Scale-Revised	Atopic dermatitis/Physician’s diagnosis (Hanifin and Rajka criteria)	Yes
Chang et al. 2013^59^	Continuous data for HI and IN	Cross-sectional	Seoul and Ilsan and Gwacheon in Gyeonggi-do province in Korea	Not reported	575	3–7 years (4.8)/51.3%	CBCL	Atopic dermatitis, allergic rhinitis, and asthma/Physician’s diagnosis and questionnaire (ISAAC questionnaire and SCORAD index)	No
Goodwin et al. 2013^60^	OR of ADHD in children with asthma*(different grades of severity [moderate*]/persistent [remission* and late onset*])	Cohort	Perth, Western Australia	Not reported	2193/390/1803	5, 8, 10, 14, and 17 years/62.56%	CBCL	Asthma/Physician’s diagnosis (Australian Asthma Handbook)	Yes
Kim et al. 2014^61^	Continuous data for impulsivity and inattention (divided attention task*)	Cohort	Korea	Not reported	1036/797/239	3–16 years (11.4)/69.88%	Computerized comprehensive attention test	Allergic rhinitis/skin prick test	Yes
Lee et al. 2014^62^	Continuous data for HI and IN*	Cross-sectional	Seoul, Korea	Not reported	160/87/73	6–13 years (8.49)/39.4%	DSM-IV	Allergic rhinitis/physician’s diagnosis (ARIA guideline)	Yes
Yang et al. 2014^63^	Continuous data for HI* and IN*	Cross-sectional	Taipei, Taiwan	Not reported	122/93/29	6–14 years (10.78)/52.2%	DSM-IV-TR	Allergic rhinitis/Physician’s diagnosis (ARIA 2008) + IgE test (MAST or Phadiatop tests)	Yes
Catal et al. 2016^64^	Continuous data for total ADHD symptoms* and percentage of psychiatric disorders determined by ECI-4*	Cross-sectional	Malatya, Samsun, Aydın, Istanbul, Turkey	Not reported	154/80/74	3–5 years (4.03)/51.3%	ECI-4	Atopic dermatitis/Physician’s diagnosis (Hanifin and Rajka criteria)	No
Hammer-Helmich et al. 2016^65^	Continuous data for total ADHD symptoms *©	Cross-sectional	Copenhagen, Denmark	Not reported	9036/2433/6603	3–15 years (NA)/49.5%	SDQ	Eczema, asthma, and hay fever/Questionnaire (UK Working Party Criteria)	Yes
Strom et al. 2016^66^	OR of ADD/ADHD in children with atopic dermatitis*	Cross-sectional	U.S. Bureau of Census	Not reported	180,799/17,277/163,522	< 18 years (NA)/49.94%	Questionnaire	Atopic dermatitis, asthma, and allergic rhinitis/questionnaire	Yes
Yang et al. 2016^67^	Continuous data for HI* and IN*	Case–control	Taipei, Taiwan	Not reported	99/68/31	6–14 years (9.25)/82.4%	DSM-IV	Allergic rhinitis/physician’s diagnosis + IgE test (MAST or Phadiatop tests)	Yes
Feng et al. 2017^68^	Continuous data for HI* and IN*	Cross-sectional	Wenzhou, China	Not reported	643/248/320	6–12 years (9.25)/51.9%	DSM-IV	Allergic rhinitis/physician’s diagnosis (ARIA 2008) + skin prick test	Yes
Lin et al. 2017^45^	OR of ADHD (inattentive/Hyperactive impulsive/combined) in children with atopic dermatitis, asthma, allergic rhinitis (fever/active)*	Cross-sectional	Taiwan	Han Chinese	2896/2235/661	9–10 years (10)/50.5%	SNAP-IV	Atopic dermatitis, asthma, and allergic rhinitis/questionnaire (ISAAC questionnaire)	Yes
Abd El-Hamid et al. 2018^69^	Prevalence of different grades of ADHD symptoms (mild, moderate, and severe) in children with or without atopic diseases*	Case–control	Cairo, Egypt	Not reported	160/100/60	6–12 years (8.54)/81.25%	DSM-IV	Allergic diseases/physician’s diagnosis, skin prick test, and total IgE enzyme immunoassay (ELISA)	No
Kuniyoshi et al. 2018^70^	Continuous data for total ADHD symptoms©*	Cross-sectional	Miyagi Prefecture, Japan	Not reported	9954/1641/8313	7–14 years (10.42)/49.9%	SDQ	Eczema/ISAAC (Eczema symptom questionnaire)	Yes
Schmitt et al. 2018^6^	Continuous data for HI and IN*	Case–control	Dresden, Germany	Not reported	89/35/45	6–12 years (9.9)/62.9%	ICD-10	Atopic dermatitis/dermatologist’s diagnosis (UK Working Party Criteria)	Yes
Cices et al. 2019^71^	Continuous data for HI and IN*	Case–control	US, Chicago, Illinois	Caucasian (31.43%) African American (22.86%) Hispanic (20%) Asian (20%) Other (5.71%)	35/17/18	6–16 years (11.07)/65.7%	Standardized Vanderbilt questionnaires	Atopic dermatitis/SCORAD	Yes
Tajdini et al. 2019^72^	Prevalence of ADHD in patients with asthma and controls*	Case–control	Tehran, Iran	Not reported	171/79/92	5–11 years (7.54)/57%	Child Symptom Inventory-4 (CSI-4)	Asthma/respiratory function test	Yes
Zhou et al. 2019^73^	Prevalence of ADHD in patients with asthma and controls*	Cross-sectional	Guiyang, China	Han (77.59%)	522/261/261	6–16 years (9.35)/61.69%	Mini-International Neuropsychiatric Interview for children and adolescents	Asthma/physician’s diagnosis (Bronchial Asthma Diagnostic and Prevention Guide for Children [2016 version])	Yes
Feng et al. 2020^74^	Continuous data for HI* and IN*	Cross-sectional	Chongqing, China	Not reported	273/89/184	6–12 years (8.3)/62.63%	SNAP-IV	Atopic dermatitis/dermatologist’s diagnosis (UK criteria)	Yes
Fuhrmann et al. 2020^75^	HR of ADD/ADHD in children with atopic dermatitis*	Cohort	Germany	Not reported	37,235/9257/27,978	From 0 to 7–9 years/51.7%	ICD-10	Atopic dermatitis/ICD-10	Yes
Guo et al. 2020^76^	Continuous data for HI (AR, AD, and asthma) and IN (AR*, AD, and asthma*)	Cohort	Kaohsiung, Taiwan	Not reported	97	6 years/54.6%	SNAP-IV	Atopic dermatitis, asthma, and allergic rhinitis/ISAAC questionnaire, physician’s diagnosis (Hanifin–Rajka criteria), and IgE test	Yes
Huang et al. 2020^77^	OR of ADD/ADHD in children with atopic dermatitis*	Cross-sectional	US Northeast (17.96%) North Central (19.33%) South (45.83%) West (16.42%) Unknown (0.47%)	Not reported	203,533/86,969/116,564	0–17 years (5.3)/51.74%	ICD-10-CM	Atopic dermatitis/IBM MarketScan Commercial Claims database (ICD-10-CM code for AD: L20*)	Yes
Jackson-Cowan et al. 2020^78^	OR of ADD/ADHD in children with atopic dermatitis*	Cross-sectional	US Northeast (15.73%) North Central (20.27%) South (36.21%) West (27.78%)	White (48.44%) Black (15.38%) Hispanic (27.86%) Asian (6.06%) Native American (0.97%) Other (1.29%)	109,482/13,398/96,084	2–17 years/51.48%	Questionnaire	Atopic dermatitis/questionnaire	Yes
Kuo et al. 2020^41^	Continuous data for HI* and IN	Cross-sectional	Kaohsiung, Taiwan	Not reported	191/109/92	(6.59)/54.44%	SNAP-IV	Atopic dermatitis, asthma, and allergic rhinitis/physician’s diagnosis	Yes
Minatoya et al. 2020^79^	Continuous data for total ADHD symptoms*	Cross-sectional	Hokkaido, Japan	Not reported	3862/799/3063	(5.28)/49.9%	SDQ	Atopic dermatitis/ISAAC	Yes
Montalbano et al. 2020^80^	Continuous data for total ADHD symptoms *	Cross-sectional	Florence, Italy	Not reported	141	6–11 years (10)/61.7%	SDQ	Asthma/GINA	Yes
Sollander et al. 2020^81^	OR of ADD/ADHD symptoms in children with atopic dermatitis*	Cross-sectional	Uppsala, Sweden	Not reported	4451/397/4054	3–5 years (4.34)/50.42%	SDQ	Asthma/questionnaire	Yes
Wan et al. 2020^82^	OR of significant ADD/ADHD symptoms in children with atopic dermatitis*	Cross-sectional	US	White (66.6%)Black (20.9%)Indian (0.9%)Asian (5.3%)Multiracial (6.3%)	57,726,856/6,807,687/50,919,169	(10.08)/48.6%	SDQ	Atopic dermatitis/questionnaire	Yes
Berzosa-Grande et al. 2021^19^	Continuous data for IN*	Cross-sectional	Madrid, Spain	Not reported	366/194/172	6–11 years (8.68)/52.7%	CBCL	Asthma, atopic dermatitis, allergic rhinitis/questionnaire	No
Chai et al. 2021^83^	OR of comorbidity of ADHD and Asthma*	Cross-sectional	Canada	Not reported	768,460/28,729/158,064	(8.7)/45.8%	ICD-10	Asthma/clinical physician’s diagnosis	No
Galéra et al. 2021^84^	Continuous data for ADHD symptoms*	Cohort	Canada	Not reported	2120	1.5, 2.5, 3.5, 4.5, 5, 6, 7, 8, 10, 13, 15, 17 years/50.9%	Behavioral ratings of ADHD symptoms	Asthma, eczema/Code of database (Quebec Longitudinal Study of Child Development (QLSCD))	Yes
Hou et al. 2021^85^	OR of ADD/ADHD in children with atopic dermatitis*	Cross-sectional	US	Not reported	228,898/23,353/205,545	< 17 years/50.2%	database	Atopic dermatitis/questionnaire	Yes
Keller et al. 2021^86^	Association between asthma, atopic dermatitis* and ADHD symptoms	Cross-sectional	Leipzig, Germany	Not reported	1764	2.53–10.49 years(6.1)/53.1%	SDQ	Asthma, atopic dermatitis/questionnaire	No
Özyurt et al. 2021^87^	Continuous data for ADHD symptoms*	Cross-sectional	Izmir, Turkey	Not reported	121/61/60	12–18 years (14.27)/47.54%	SDQ	Asthma/GINA (2017)	No
Rajhans et al. 2021^88^	Continuous data for IN*	Cross-sectional	North India	Not reported	60/30/30	8–15 years (11.92)/70%	M.I.N.I. KID, CBCL	Asthma/GINA (2015)	Yes
Vittrup et al. 2021^89^	HR of ADHD in children with atopic dermatitis*	Cohort	Denmark	Not reported	157,113/14,283/142,830	0–18 years/57%	Codes of database	Atopic dermatitis/clinical physician’s diagnosis	Yes
Yüksel et al. 2021^90^	Continuous data for HI* and IN in ADHD children with or without asthma	Cross-sectional	Izmir, Turkey	Not reported	355/91/264	2–6 years	ADHD Rating Scale-IV, K-SADS-PL	Asthma/questionnaire	Yes

ADHD, Attention-Deficit/Hyperactivity Disorder; OR, Odds Ratio; HI, Hyperactivity/Impulsivity; IA, Inattention; DSM-IV, Diagnostic and Statistical Manual of Mental Disorders; GINA 2006, Global Initiative for Asthma, 2006; CBCL, Child Behavior Checklist; ARIA, Allergic Rhinitis and its Impact on Asthma; MAST, multiple-antigen simultaneous test; ECI-4, The Early Childhood Inventory-4; ADD, Attention deficit disorder; SDQ, Strengths and Difficulties Questionnaire; ICD-10, International Classification of Diseases, Tenth Revision; SCORAD, SCORing Atopic Dermatitis; ISAAC, International Study of Asthma and Allergies in Childhood; M.I.N.I. KID, Mini-International Neuropsychiatric Interview for Children and Adolescents; K-SADS-PL; Schedule for Affective Disorders and Schizophrenia for School-Aged Children—Present and Lifetime Version © Calculated from the available information in the article. *Statistically significant.

Three of the four studies reported a significant association between the comorbidity of ADHD and atopic diseases in patients with ADHD. Ten studies reported a significantly positive correlation among atopic diseases, sleep disturbance, and ADHD or an increased risk of ADHD in children with atopic diseases. One research group^26^ performed structural equation modeling to determine interactions among eczema, asthma, rhinitis, sleep, and behavior disorders. In three studies, the computerized comprehensive attention test (CAT) was performed, a more objective and precise measurement tool, to assess attention deficit, and they obtained results similar to those of other studies, thus supporting our hypothesis that atopy increased ADHD symptoms severity. In addition, eight studies examined treatment modalities for atopic diseases; however, additional randomized controlled trials are required to obtain more consistent results and causal relationships. The included studies evaluated several confounding factors such as age, sex, and atopic disease severity. Additional detailed information is provided in Table S1 in the Supplementary Materials.

### Quantitative review and meta-analysis of cross-sectional studies

Sixteen studies including 25,337 participants examined the relationship between at least one type of atopic disease and the severity of total ADHD symptoms, hyperactivity/impulsivity or inattention, by including unexposed groups consisting of participants without atopic diseases. Table 2 lists the characteristics of included studies. Of the included studies, all studies were cross-sectional designs.

**Table 2:** Studies examining the relationship between atopic diseases and hyperactivity in the meta-analysis.

Study name	Country	Study design	Number of participants/Exposed group/unexposed group	Age (mean of the exposed group)/sex (male, %)	Diagnostic criteria for ADHD	Atopic disease/method used for assessing atopic diseases	Outcome scale	Overall risk of bias
Yuksel et al. 2008^58^	Turkey	Cross-sectional	100/62/38	7–12 years (9.2)/57%	DSM-IV	Asthma/physician’s diagnosis (GINA 2006)	Conner’s Parent Rating Scale	Included
Camfferman et al. 2010^26^	Australia	Cross-sectional	107/77/30	6–16 years (9.9)/46.72%	Conner’s Parent Rating Scale-Revised	Atopic dermatitis/physician’s diagnosis (Hanifin and Rajka criteria)	Conner’s Parent Rating Scale	Included
Lee et al. 2014^62^	Republic of Korea	Cross-sectional	160/87/73	6–13 years (8.49)/39.4%	DSM-IV	Allergic rhinitis/physician’s diagnosis (ARIA guideline)	ARS	Included
Yang et al. 2014^63^	Taiwan	Cross-sectional	122/93/29	6–14 years (10.78)/52.2%	DSM-IV-TR	Allergic rhinitis/physician’s diagnosis (ARIA 2008) + IgE test (MAST or Phadiatop tests)	SNAP-IV (parent)	Included
Catal et al. 2016^64^	Turkey	Cross-sectional	154/80/74	3–5 years	ECI-4	Atopic dermatitis/physician’s diagnosis (Hanifin and Rajka criteria)	ECI-4	Included
Hammer-Helmich et al. 2016^65^	Denmark	Cross-sectional	9036/2433/6603	3–15 years (NA)/49.5%	SDQ	Eczema, asthma, and hay fever/Questionnaire	SDQ	Included
Yang et al. 2016^67^	Taiwan	Case–control	99/68/31	6–14 years (9.25)/82.4%	DSM-IV	Allergic rhinitis/Physician’s diagnosis + IgE test (MAST or Phadiatop tests)	SNAP-IV (parent)	Included
Feng et al. 2017^68^	China	Cross-sectional	643/248/320	6–12 years (9.25)/51.9%	DSM-IV	Allergic rhinitis/Physician’s diagnosis (ARIA 2008) + skin prick test	SNAP-IV	Included
Kuniyoshi et al. 2018^70^	Japan	Cross-sectional	9954/1641/8313	7–14 years (10.42)/49.9%	SDQ	Eczema/ISAAC Eczema Symptom questionnaire	SDQ	Included
Schmitt et al. 2018^6^	Germany	Case–control	89/35/45	6–12 years (9.9)/62.9%	ICD-10	Atopic dermatitis/Dermatologist’s diagnosis (UK Working Party Criteria)	FBB-HKS	Included
Feng et al. 2020^74^	China	Cross-sectional	273/89/184	6–12 years (8.3)/62.63%	SNAP-IV	Atopic dermatitis/Dermatologist’s diagnosis (UK criteria)	SNAP-IV	Included
Kuo et al. 2020^41^	Taiwan	Cross-sectional	191/109/92	(6.59)/54.44%	SNAP-IV	Atopic dermatitis, asthma, and allergic rhinitis/Physician’s diagnosis	SNAP-IV	“Seek further information”
Minatoya et al. 2020^79^	Japan	Cross-sectional	3862/799/3063	(5.28)/49.9%	SDQ	Atopic dermatitis/ISAAC	SDQ	Included
Berzosa-Grande et al. 2021^19^	Spain	Cross-sectional	366/194/172	6–11 years (8.68)/52.7%	CBCL	Asthma, atopic dermatitis, allergic rhinitis/questionnaire	CBCL	Included
Özyurt et al. 2021^87^	Turkey	Cross-sectional	121/61/60	12–18 years (14.27)/47.54%	SDQ	Asthma/GINA (2017)	SDQ	Included
Rajhans et al. 2021^88^	India	Cross-sectional	60/30/30	8–15 years (11.92)/70%	M.I.N.I. KID, CBCL	Asthma/GINA (2015)	CBCL	Included

ADHD, Attention-Deficit/Hyperactivity Disorder; DSM-IV, Diagnostic and Statistical Manual of Mental Disorders; GINA 2006, Global Initiative for Asthma, 2006; ARIA, Allergic Rhinitis and its Impact on Asthma; ARS, ADHD Rating Score; MAST, multiple-antigen simultaneous test; ECI-4, The Early Childhood Inventory-4; SNAP-IV, Swanson, Nolan, and Pelham Questionnaire; SDQ, Strengths and Difficulties Questionnaire; CBCL, Child Behavior Checklist; M.I.N.I. KID, Mini-International Neuropsychiatric Interview for Children and Adolescents; ICD-10, International Classification of Diseases, Tenth Revision; FBB-HKS, The German ADHD Rating Scale.

### Overview of total ADHD symptoms through a meta-analysis

**Figure 1:**
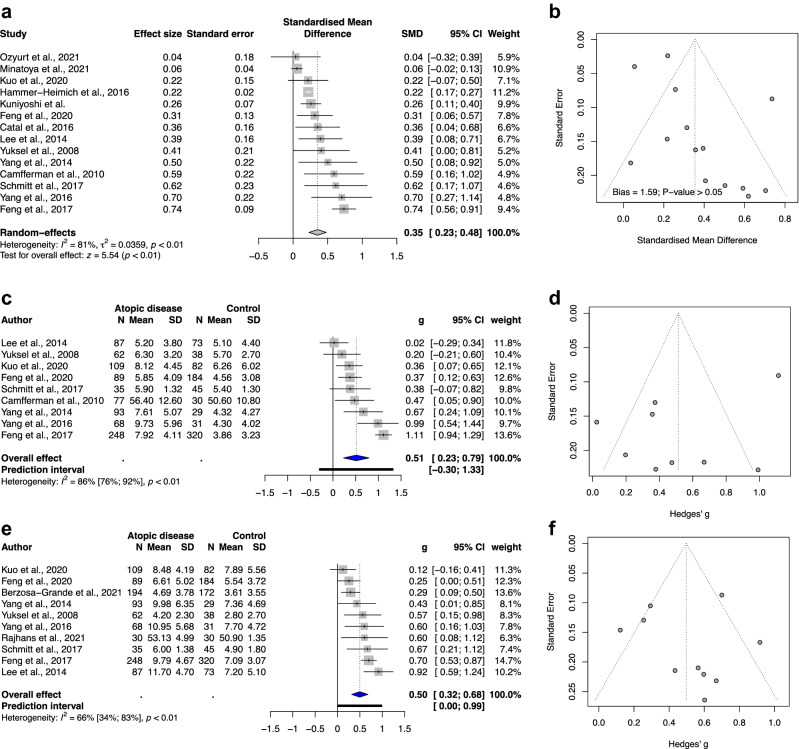
(**a**) Forest plot of the meta-analysis of the severity of total ADHD symptoms in childhood and the presence of atopic diseases. (**b**) Funnel plot of the meta-analysis of the severity of total ADHD symptoms in childhood and the presence of atopic diseases. (**c**) Forest plot of the meta-analysis of the severity of hyperactivity/impulsivity in childhood and the presence of atopic diseases. (**d**) Funnel plot of the meta-analysis of the severity of hyperactivity/impulsivity in childhood and the presence of atopic diseases. (**e**) Forest plot of the meta-analysis of the severity of inattention in childhood and the presence of atopic diseases. (**f**) Funnel plot of the meta-analysis of the severity of inattention in childhood and the presence of atopic diseases. Abbreviations: SD, standard deviation; CI, confidence interval; SMD, standardized mean difference.

In terms of the outcome of total ADHD symptoms, the overall effect exhibited a statistically significant association with standardized mean difference (SMD) of 0.35, 95% CI of 0.23–0.48, and *p* value < 0.01 (Fig. 1a). We observed considerable heterogeneity among subgroups with an *I*^2^ value of 81%. The funnel plot displayed no asymmetry (*p* > 0.05; Fig. 1b).

### Overview of hyperactivity/impulsivity through a meta-analysis

In terms of the outcome of hyperactivity/impulsivity, the overall effect showed a statistically significant association with atopic diseases, with SMD of 0.51, 95% CI: 0.23–0.79, and *p* value of 0.003 (Fig. 1c). Substantial to considerable heterogeneity was noted (*I*^2^ value: 86%). The funnel plot (Fig. 1d) and Egger’s test (Figure S1; *p* = 0.15) showed no significant publication bias.

### Overview of inattention through a meta-analysis

The overall effect of the outcome of inattention showed a significant and positive correlation with atopic diseases, with SMD of 0.5, 95% CI: 0.32–0.68, and *p* value of 0.0002 (Fig. 1e). Different from the outcome of hyperactivity/impulsivity, substantial heterogeneity (*I*^2^ = 66%) was observed for the outcome of inattention. The funnel plot (Fig. 1f) and Egger’s test (Figure S2; *p* = 0.88) showed no statistically significant publication bias.

### Meta-analysis of ADHD-related behavioral problems in atopic children without previous ADHD diagnosis

**Figure 2:**
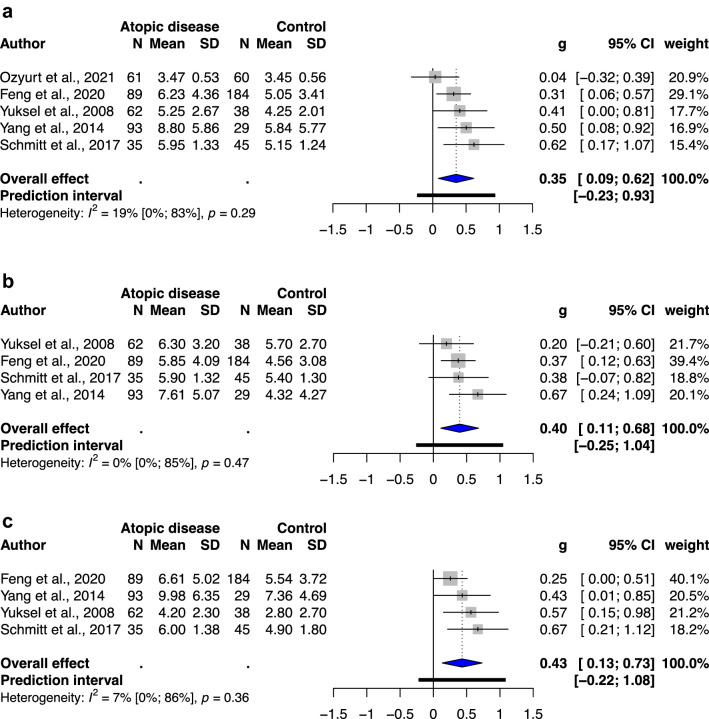
(**a**) Forest plot of the subgroup analysis of the severity of total ADHD symptoms in childhood and the presence of atopic diseases. (**b**) Forest plot of the subgroup analysis of the severity of hyperactivity/impulsivity in childhood and the presence of atopic diseases. (**c**) Forest plot of the subgroup analysis of the severity of inattention in childhood and the presence of atopic diseases. Abbreviations: CI, confidence interval; SMD, standardized mean difference.

Five, four, and four studies included an atopic group without previous ADHD diagnosis and a healthy unexposed group to examine the outcomes of ADHD-related behavioral problems, which indicated total ADHD symptoms, hyperactivity/impulsivity, and inattention, respectively. All studies investigating total ADHD symptoms, hyperactivity/impulsivity, and inattention exhibited a statistically significant association, with SMD of 0.35(95% CI: 0.09–0.62, *p* value: 0.0212 (Fig. 2a)), SMD of 0.40(95% CI: 0.11–0.68, *p* value: 0.0212(Fig. 2b)), and SMD of 0.43(95% CI: 0.13–0.73, *p* value: 0.0193(Fig. 2c)), respectively. Low heterogeneity was noted in this meta-analysis (*I*^2^ = 0–19%).

### Subgroup analysis of different types of atopic diseases

**Figure 3:**
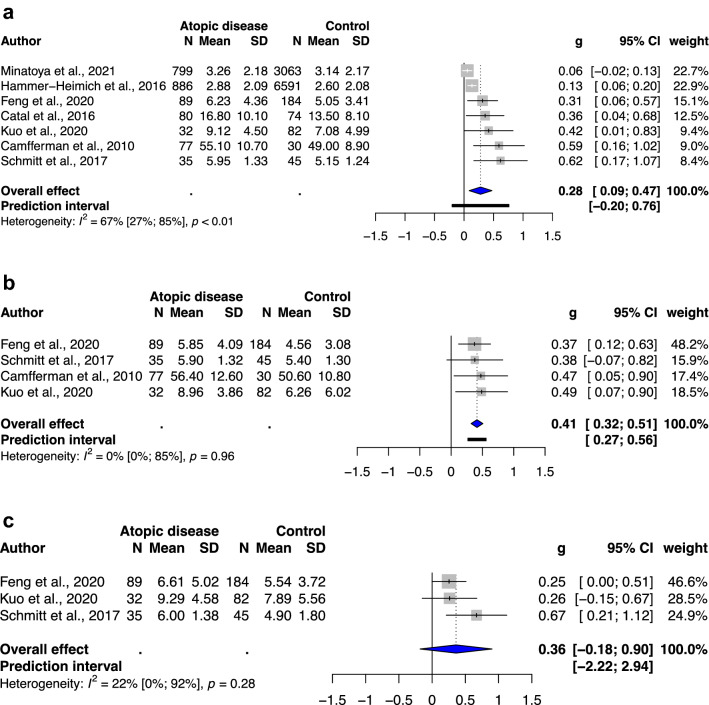
(**a**) Forest plot of the subgroup analysis of the severity of total ADHD symptoms in children with atopic dermatitis. (**b**) Forest plot of the subgroup analysis of the severity of hyperactivity/impulsivity in children with atopic dermatitis. (**c**) Forest plot of the subgroup analysis of the severity of inattention in children with atopic dermatitis. Abbreviations: CI, confidence interval; SMD, standardized mean difference.

**Figure 4:**
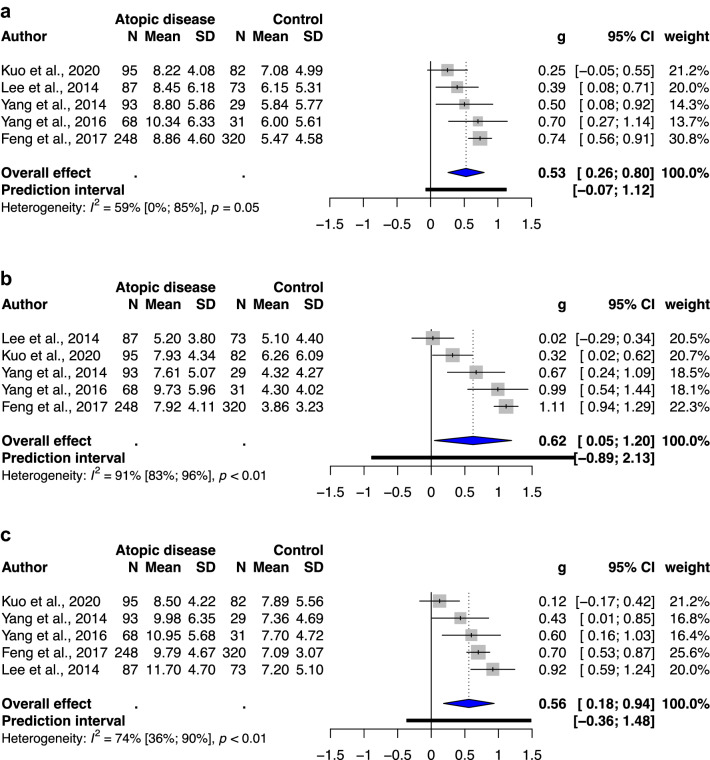
(**a**) Forest plot of the subgroup analysis of the severity of total ADHD symptoms in children with allergic rhinitis. (**b**) Forest plot of the subgroup analysis of the severity of hyperactivity/impulsivity in children with allergic rhinitis. (**c**) Forest plot of the subgroup analysis of the severity of inattention in children with allergic rhinitis. Abbreviations: CI, confidence interval; SMD, standardized mean difference.

**Figure 5:**
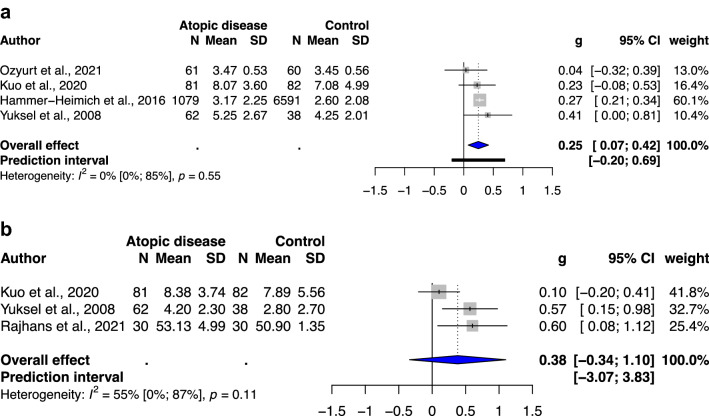
(**a**) Forest plot of the subgroup analysis of the severity of total ADHD symptoms in children with asthma. (**b**) Forest plot of the subgroup analysis of the severity of hyperactivity/impulsivity in children with asthma. (**c**) Forest plot of the subgroup analysis of the severity of inattention in children with asthma. Abbreviations: CI, confidence interval; SMD, standardized mean difference.

A significant association was observed between atopic dermatitis and total ADHD symptoms (SMD: 0.28, 95% CI: 0.09–0.47, *p* value: 0.012) and hyperactivity/impulsivity (SMD: 0.41, 95% CI: 0.32–0.51, *p* value: 0.0008), with low to moderate heterogeneity (*I*^2^ = 0–67%; Fig. 3). A significant association was noted between allergic rhinitis and total ADHD symptoms (SMD: 0.53, 95% CI: 0.26–0.80, *p* value: 0.006), hyperactivity/impulsivity (SMD: 0.62, 95% CI: 0.05–1.20, *p* value: 0.04), and inattention (SMD: 0.56, 95% CI: 0.18–0.94, *p* value: 0.015), with moderate to substantial heterogeneity (I^2^ = 59–91%; Fig. 4). Furthermore, a significant association was observed between asthma and only total ADHD symptoms (SMD: 0.25, 95% CI: 0.07–0.42, *p* value: 0.02), with low to substantial heterogeneity (*I*^2^ = 0–55%; Fig. 5). The analysis of hyperactivity in children with asthma was not performed due to insufficient studies (n = 2).

As sensitivity analysis, we observed decreased heterogeneity in meta-analysis of ADHD-related behavioral problems in children without previous ADHD diagnosis. However, in subgroups were categorized according to the types of atopic diseases, only the atopic dermatitis and asthma subgroup showed decreased heterogeneity (*I*^2^ = 0–67%), whereas allergic rhinitis subgroup showed low to considerable heterogeneity.

## Discussion

The results of our study indicated that atopic diseases were related to not only increased odds of ADHD but also the severity of ADHD symptoms. Total ADHD symptoms, hyperactivity/impulsivity, and inattention were all significantly associated with atopic diseases. The results of the subgroup analysis indicated that children with subthreshold ADHD in the atopic disease groups had increased severity of ADHD symptoms, including total ADHD symptoms, hyperactivity/impulsivity, and inattention, and atopic dermatitis and allergic rhinitis were significantly associated with ADHD symptoms.

Although we included a small number of studies, these studies in total included more than 25,000 participants. In addition, this is the first systematic review and meta-analysis to investigate not only the association of atopic diseases with the severity of ADHD symptoms but also the presentation of ADHD-related behavioral symptoms in atopic children without previous ADHD diagnosis. The results of both the qualitative systematic review and meta-analysis tended to demonstrate higher severity of total ADHD symptoms and inattention in participants with atopic diseases, and the meta-analysis showed a significant association between atopic diseases and hyperactivity-impulsivity severity although qualitative synthesis showed relatively inconsistent outcome. Three studies that adopted the computerized CAT to assess attention deficits also demonstrated similar results, thus supporting our hypothesis. However, our qualitative systematic review and meta-analysis exhibited inconsistency in outcomes and considerable heterogeneity.

We performed a second meta-analysis of ADHD-related behavioral problems in atopic children without previous ADHD diagnosis, which not only consisted of studies with more strict exclusion criteria for diagnosed ADHD, but also reflected on the association between atopic diseases and subthreshold ADHD. The result showed statistical significance with low heterogeneity, indicating that atopic children was associated with subthreshold ADHD, as ADHD-related behavioral problems, despite of lack of diagnosed ADHD. The subgroup analysis of different types of atopic diseases showed that atopic dermatitis was significantly and positively correlated with the severity of total ADHD symptoms and hyperactivity/impulsivity, with low to moderate heterogeneity (*I*^2^ = 0–67%). Allergic rhinitis was significantly associated with the severity of total ADHD symptoms, hyperactivity/impulsivity, and inattention, with moderate to considerable heterogeneity (*I*^2^ = 59–91%). Asthma was significantly associated only with the severity of total ADHD symptoms, with low to substantial heterogeneity (*I*^2^ = 0–55%).

This inconsistency in heterogeneity can be attributed to differences in participants’ sex and mean age and the inclusion and exclusion criteria of related studies. A previous study reported a higher male to female ratio for hyperactivity but an equal male to female ratio for inattention^27^. This study also indicated that the distribution of sex differences within the severity of symptoms varied between children without ADHD and children with ADHD. The differences in the clinical course of participants can also result in these inconsistencies. Hyperactivity/impulsivity is usually observed from 4 years of age, peaks in severity at approximately 7 to 8 years, and then declines later, whereas inattention is not apparent until 8–9 years of age^28^. In this study, we observed a significantly decreased heterogeneity in the meta-analysis of ADHD-related behavioral problems in atopic children without previously diagnosed ADHD, indicating that inconsistent exclusion criteria for ADHD may have been the source of heterogeneity in our study. Moreover, the lack of standard inclusion and exclusion criteria for not only ADHD but also other neuropsychiatric disorders or developmental delays may be responsible for the high heterogeneity in our study. Furthermore, our results suggested that children with atopic diseases without previous ADHD diagnosis had a stronger association of more severe core symptoms of ADHD than healthy unexposed groups, indicating a more consistent result for inattention than hyperactivity/impulsivity, with remarkably decreased heterogeneity.

We also observed low to considerable heterogeneity in the subgroup analysis of different types of atopic diseases. Asthma had a significant association only with the severity of total ADHD symptoms, not with inattention. Considering that previous studies have reported that children with asthma had an increased risk of developing severe ADHD symptoms^18,22,29^, this result may be attributed to the small number of included studies (n = 3). The results of the subgroup analysis of different types of atopic diseases indicated that all atopic diseases were significantly associated with the severity of total ADHD symptoms; however, inconsistent heterogeneity was observed in the association between atopic diseases and the severity of hyperactivity/impulsivity and inattention. These findings suggest that different types of atopic diseases may have distinct interactions with ADHD symptoms, although our results demonstrated that any type of atopic disease was associated with the severity of ADHD symptoms.

Although the causal relationship and mechanism underlying the association between atopic diseases and ADHD remain unclear, many studies have provided several hypotheses^30^) (Figure S3). One of the more commonly accepted hypotheses is that atopic diseases induce inflammation, including Th1, Th2, and Th17 immune responses, which lead to the downstream hypersecretion of IgE^31–33^ (Figure S4). These inflammatory factors may affect the neuroactivity of the prefrontal cortex (PFC) and anterior cingulate cortex (ACC) that have been reported to be strongly associated with ADHD pathology^34–36^. This process affects the maturation of the PFC and ACC, which usually occurs in early life when the brain is undergoing profound changes and is important for the development of cognitive function^37^. In addition to the direct effect, cytokines may indirectly affect these brain areas by disturbing the hypothalamic–pituitary–adrenal axis (HPA) axis^38^, altering the central metabolism of neurotransmitters including norepinephrine and dopamine^35,39^.

In addition to neuroimmunological pathways, psychological mechanisms should also be considered. Atopic diseases cause psychological stress in not only patients but also their main caregivers since early childhood when the onset of atopic diseases occurs^40,41^. An unsatisfactory parent–child relationship characterized by overprotection, anxiety, low support, and poor sleep quality can lead to a decline in psychosocial and cognitive performance due to tiredness; emotional problems were reported to be associated with negative outcomes in a previous study^42^. Although the relationship between these effects and brain development in early life has not yet been well studied, stress in early childhood is believed to affect the balance between neurotransmitters and neuroendocrine systems, including norepinephrine, dopamine, and the HPA axis^38,43^. This eventually resulted in increased vulnerability to psychological diseases, such as ADHD, because of altered neuropsychologic pathways leading to unsuccessful brain development and maturation^44^.

An increased risk of the development and progression of atopic diseases was observed in children with ADHD because atopic diseases are attributed to stress^45^. Although these mechanisms were obscure, studies have found that stress may induce deficits in skin barriers, and a similar inflammatory cascade with atopy-associated immune responses was observed in patients with atopic diseases; some patients exhibited an increased tendency to exhibit Th1 responses^46^. These findings indicate that stress can exacerbate atopic diseases. ADHD was reported to be related to psychosocial stress because poor family support, school performance, and peer relationships can all be the sources of stress^47^. Therefore, ADHD-related stress can exacerbate atopic diseases. Recently, genetic factors and prenatal stress have been indicated as common risk factors for atopic diseases and ADHD. Although few studies have evaluated genetic susceptibility and other associated interaction factors leading to epigenetic reactions^48,49^, a twin study supported the hypothesis that common genetic factors between atopy and ADHD exist^50^. Moreover, studies reporting a relationship between maternal stress and symptoms of atopic diseases and ADHD have indicated that dysregulation of the HPA axis results in delayed brain development and a shift in Th1/Th2 balance, resulting in an atopic disease–prone immune profile^51,52^.

Previous systematic reviews and meta-analyses had discussed about increased risk of diagnosed ADHD in atopic group; however, the evidence of whether atopic diseases associated with increased severity of ADHD symptoms in participants with subthreshold ADHD remained unclear. This association could be observed in the meta-analysis of ADHD-related behavioral problems in atopic children without previously diagnosed ADHD in the present study, provided an evidence that atopic diseases are associated with “spectrum of ADHD symptom severity”, the term which we quoted following a previous study to explain how atopic diseases associated with ADHD symptoms^53^, and this association existed in subthreshold ADHD group in our result as well.

### Limitations

This study has several limitations that should be considered. First, although more than 25,000 participants were included, the outcome of hyperactivity was examined in only 1700 participants, which may reduce the strength of evidence. Second, Atopic diseases were diagnosed based on physicians’ decisions or by using some questionnaires in the included studies; these might have caused heterogeneity. Third, we chose to combine scores of hyperactivity/impulsivity and inattention as the primary outcome, and this method has been used in several studies^54^. However, potential systematic errors may still occur. Forth, differences in exclusion criteria, including the exclusion of patients with ADHD and other neuropsychiatric disorders and development problems, caused significant heterogeneity in our study. Finally, some studies included children with multiple atopic diseases, which may be potential confounding factors in our study.

To minimize the effect of those limitations, we conducted sensitivity analysis and subgroup analysis to assess the effects of potential confounding factors, namely different types of atopic diseases and exclusion criteria for previous diagnosed ADHD and other neuropsychiatric disorders; this considerably reduced heterogeneity in sensitivity analysis and most subgroup analyses. Therefore, standardized and more strictly defined exclusion and inclusion criteria should be included in future studies. Moreover, additional studies investigating the relationship between different types of atopic diseases and ADHD symptoms should be conducted because we could not conduct a complete subgroup analysis of different atopic diseases owing to the limited number of studies.

## Conclusion

Our study results indicated that atopic diseases not only increased the odds of ADHD but also were associated with more severe core symptoms of ADHD. We observed increased severity of ADHD-related behavioral symptoms in children with atopic diseases without previously diagnosed ADHD, indicating that atopic diseases may also associated with spectrum of ADHD symptom severity in participants with subthreshold ADHD, which was never been investigated in previous researches. According to the results of this study, while treating children with ADHD, clinicians should consider the possibility of comorbid atopic diseases. On the other hand, the comorbidity of ADHD should be considered when treating children with atopic diseases. Moreover, clinicians should be aware of the increased ADHD-related behavioral symptoms in children with atopic diseases. Additional studies including more strictly defined criteria, studies investigating the mechanism underlying this association, and randomized controlled trials of related therapeutic strategies should be conducted.

## Methods

This systematic review and meta-analysis was conducted in accordance with the Preferred Reporting Items for Systematic Reviews and Meta-Analyses (PRISMA) guidelines^55^. Related checklists are provided in the Supplementary Materials. Two researchers searched for and pooled observational studies examining atopic diseases and ADHD symptom severity to examine the association between them.

### Eligibility criteria for study selection

To reduce selection bias, we defined eligibility criteria before the inclusion of studies. We included the following studies: (1) observational studies investigating the association between one of the three major types of atopic diseases (i.e., asthma, eczema, and allergic rhinitis) and the severity of ADHD symptoms, irrespective of whether they included patients with ADHD in the exposed group; (2) studies recruiting children and adolescents; (3) studies including any of the three atopic diseases as the exposure variable and ADHD symptom severity (scores of a behavior rating scale) as the outcome variable; (4) studies investigating the severity of ADHD symptoms in patients with at least one type of atopic disease by using any type of assessment method; (5) studies including a matched or an unmatched unexposed group for comparison with the exposed group; and (6) cross-sectional, case–control, or cohort studies. The following studies were excluded: (1) studies that did not provide adequate information regarding the relationship between atopic diseases and ADHD symptom severity, including the crude data of exposed cases and the outcome of the association between at least one type of atopic disease and the severity of hyperactivity/impulsivity, inattention, and/or total ADHD symptoms (hyperactivity/impulsivity + inattention) determined on the basis of the scores of a behavior rating scale; (2) studies including only outcomes measured after interventions or those not including a separate outcome of total ADHD symptoms, hyperactivity/impulsivity, and inattention; (3) studies using the same database in different published articles (studies with longer follow-up periods and higher quality, which were classified as included (see ‘Table S2 in the Supplementary Materials’) were included); and (4) studies whose full texts were not available or those for which only abstracts or editorial materials (i.e., comments, responses, and letters without original data) were available.

### Search strategy and study selection

Two authors performed a literature search on PubMed (Medline), Embase, and Psycinfo for studies published up to December 18, 2021. The following search terms were combined and adjusted to fulfill the demand of the database.

- “Atopic eczema” [MeSH] OR “atopic dermatitis” [MeSH] OR “asthma” [MeSH] OR “allergic rhinitis” [MeSH]
- “ADHD” [MeSH] OR “attention deficit and hyperactivity disorder” [MeSH] OR “hyperactivity” [MeSH] OR “inattention” [MeSH] OR “attention deficit” [Mesh] OR “impulsivity’ [Mesh] OR “mental health” [MeSH] OR “behavior problem” [MeSH]
- “Preschool children” OR “preschooler” OR “children” OR “adolescent” OR “toddler”

A three-step screening strategy was adopted. In the title screening step, studies that met the inclusion criteria and were not letters or replies were included. In the abstract screening step, studies that were original research and included relevant unexposed groups were included. In the full-text screening step, studies for which full texts and data were available and those that did not meet the exclusion criteria were finally included. A third author resolved any disagreement between the two authors in terms of the eligibility and inclusion of studies through discussion.

### Data extraction

By using a customized data form, the first author extracted the following information from eligible studies: study title, study design, study characteristics (country, composition of participants, and outcome assessment scales used), and assessment methods and criteria for diagnosing atopic diseases and ADHD. Subsequently, the second author reviewed the extracted data. Any disagreement regarding data extraction was resolved by the third author through discussion. If required, data were calculated from the available data of included studies (see ‘Data abstraction for the meta-analysis’). The main outcome variables were the scores of the behavior rating scales used to evaluate hyperactivity/impulsivity and inattention in the atopic disease and unexposed groups. Although the included studies used different outcome scales, they were all based on the Diagnostic and Statistical Manual of Mental Disorders, 4th Edition (DSM-IV) criteria. In addition, to minimize the potential confounding effects, we analyzed standardized data. Because of the limited availability of studies, we combined the three types of atopic diseases in the meta-analysis and conducted a subgroup analysis. The authors individually recorded abstracted data in Microsoft Excel.

### Data abstraction for the meta-analysis

The relationship between one of the three main types of atopic diseases with the severity of hyperactivity/impulsivity, inattention, or total ADHD symptoms was independently investigated. To exclude confounding factors, any study including outcomes measured after interventions were excluded. For each study, the relative weight of outcome data to the whole data set was calculated using the generic inverse variance method along with the 95% confidence interval (95% CI) by using Rstudio software, Version 1.2.5042 Rstudio^56^. The random effects model with the Hartung–Knapp–Sidik–Jonkman method was used to determine the estimated variance for pooling effect sizes in our meta-analysis, with standardized mean difference as effect size. The *I*^2^ value was calculated to assess statistical heterogeneity, and this value represents the total variance of pooled data explained by the heterogeneity. According to the Cochrane handbook for systematic reviews of interventions, the *I*^2^ values of 0–40%, 30–60%, 50–90%, and 75–100% indicated low, moderate, substantial, and considerable heterogeneity^57^. We used a funnel plot to assess the potential publication bias.

For the outcome “severity of total ADHD symptoms,” if a study reported ADHD symptoms in “severity of hyperactivity/impulsivity” and “severity of inattention” separately, we calculated weighted mean and pooled standard deviation based on relevant formula before meta-analysis^57^.

For studies including a subgroup analysis, we pooled effect sizes two times. First, we combined subgroups within studies to pool effect sizes and calculated 95% CIs and standard errors. Subsequently, we included the calculated standard errors into our meta-analysis and pooled effect sizes a second time.

### Sensitivity analysis

To assess the strength of the association between atopic diseases and ADHD in different scenarios, we performed a sensitivity analysis by investigating patients with strictly defined exclusion criteria for previous ADHD diagnosis. We included studies researching both exposed and unexposed groups with exclusion criteria for previous ADHD diagnosis for a second meta-analysis, which was mentioned as meta-analysis of ADHD-related behavioral problems in atopic children without previous ADHD diagnosis. Detailed information on the risk of bias assessment could be found in Table S2 in the Supplementary Materials.

### Subgroup analysis of different type of atopic diseases

We conducted subgroup analysis of different type of atopic diseases in three outcomes: total ADHD symptoms, hyperactivity, and inattention, using a random effect model. If there were less than 3 studies included in each subgroup, we would not perform analysis.

## Supplementary Information

Supplementary Information.

## Data Availability

The datasets generated during and/or analyzed during the current study are available from the corresponding author on reasonable request.
