# Supplementary material for: Two meta-analyses of the association between atopic diseases and core symptoms of attention deficit hyperactivity disorder
Source: Sci Rep. 2022 Mar 1;12:3377. doi: 10.1038/s41598-022-07232-1 (PMC8888762; doi:10.1038/s41598-022-07232-1)
Supplement: Supplementary file 1 — Supplementary Information. [file 41598_2022_7232_MOESM1_ESM.pdf]

Supplementary Material (Appendices)

Content

Search process

Appendix A. Study search and inclusion strategy
Appendix B. PRISMA flowchart

Figure

Figure S1. Linear regression model of the Egger's test for the meta-analysis of the severity of hyperactivity/impulsivity in childhood and the presence of atopic diseases
Figure S2. Linear regression model of the Egger's test for the meta-analysis of the severity of inattention in childhood and the presence of atopic diseases
Figure S3. The mechanism of interaction between atopic diseases and ADHD
Figure S4. The comparison of immune responses and related effects between acute and chronic phase of atopic diseases

Table

Table S1. Detailed information of studies included in the qualitative analysis
Table S2. Risk of bias assessment

Others

Appendix C. References to articles solely included in qualitative synthesis
Appendix D. References to articles solely included in meta-analysis
Appendix E. Reference to articles included in supplementary materials

Search process

Appendix A. Search strategy

Primary search steps:

#1. Atopic dermatitis
#2. Atopic eczema
#3. Allergic rhinitis
#4. Asthma
#5. #1, #2, #3, OR #4
#6. ADHD
#7. Attention-deficit/hyperactivity disorder
#8. Hyperactivity
#9. Inattention
#10. Attention deficit
#11. Impulsivity
#12. Behavior problem
#13. (Mental illness) OR (Mental disorder)
#14. #6, #7, #8, #9, #10, #11, #12, #13
#15. Preschool children
#16. Preschooler
#17. Children
#18. Adolescent
#19. Toddler
#20. #14, #15, #16, #17, #18 OR #19
#21. #5 AND #14 AND #20

Final syntax in PubMed (an example):

((Atopic dermatitis) OR (Atopic eczema) OR (Allergic rhinitis) OR (Asthma)) AND ((ADHD) OR (Attention-deficit/hyperactivity disorder) OR (Hyperactivity) OR (Inattention) OR (Attention deficit) OR (Impulsivity) OR (Behavior problem) OR ((Mental illness) OR (Mental disorder))) AND ((Preschool children) OR (Preschooler) OR (Children) OR (Adolescent) OR (Toddle

Appendix B. PRISMA flowchart

PRISMA 2020 flow diagram for new systematic reviews which included searches of databases, registers and other sources

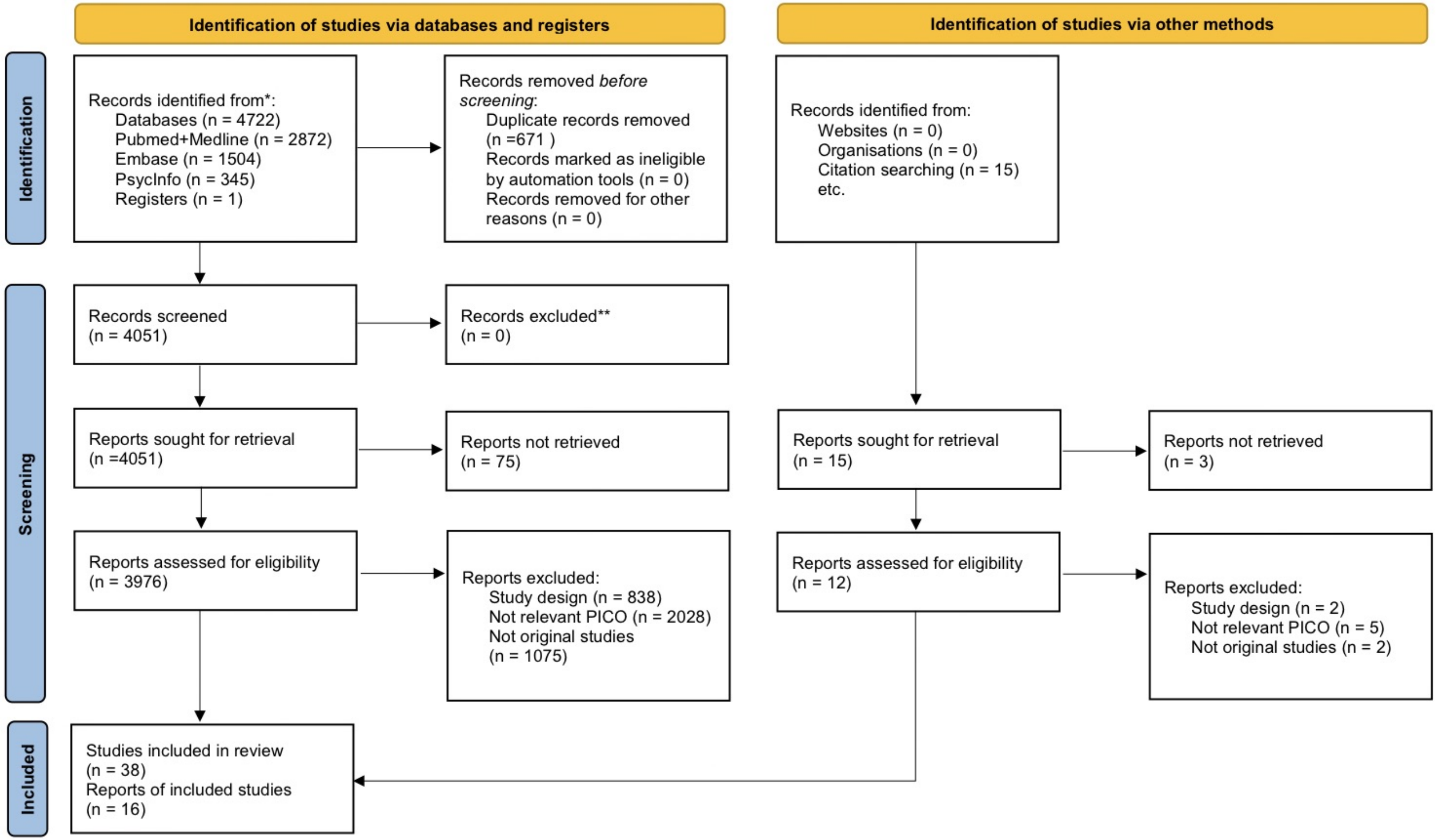

*Consider, if feasible to do so, reporting the number of records identified from each database or register searched (rather than the total number across all databases/registers).
**If automation tools were used, indicate how many records were excluded by a human and how many were excluded by automation tools.

From: Page MJ, McKenzie JE, Bossuyt PM, Boutron I, Hoffmann TC, Mulrow CD, et al. The PRISMA 2020 statement: an updated guideline for reporting systematic reviews. BMJ 2021;372:n71. doi: 10.1136/bmj.n71. For more information, visit: http://www.prisma-statement.org/

Figure

Figure S1. Linear regression model of the Egger's test for the meta-analysis of the severity of hyperactivity/impulsivity in childhood and the presence of atopic diseases

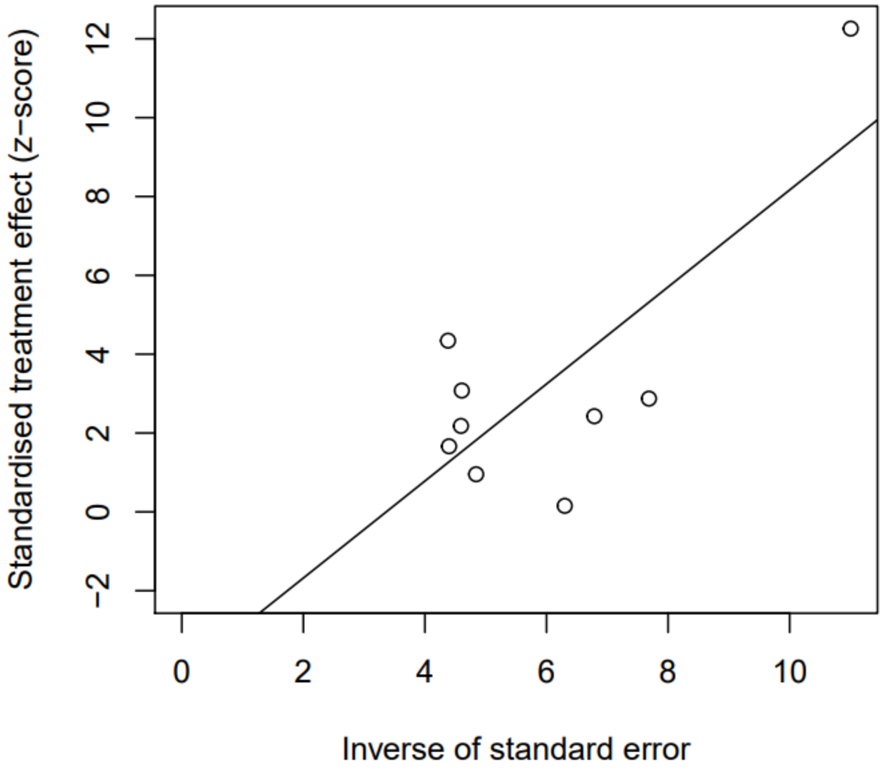

Figure S2. Linear regression model of the Egger's test for the meta-analysis of the severity of attention deficit in childhood and the presence of atopic diseases

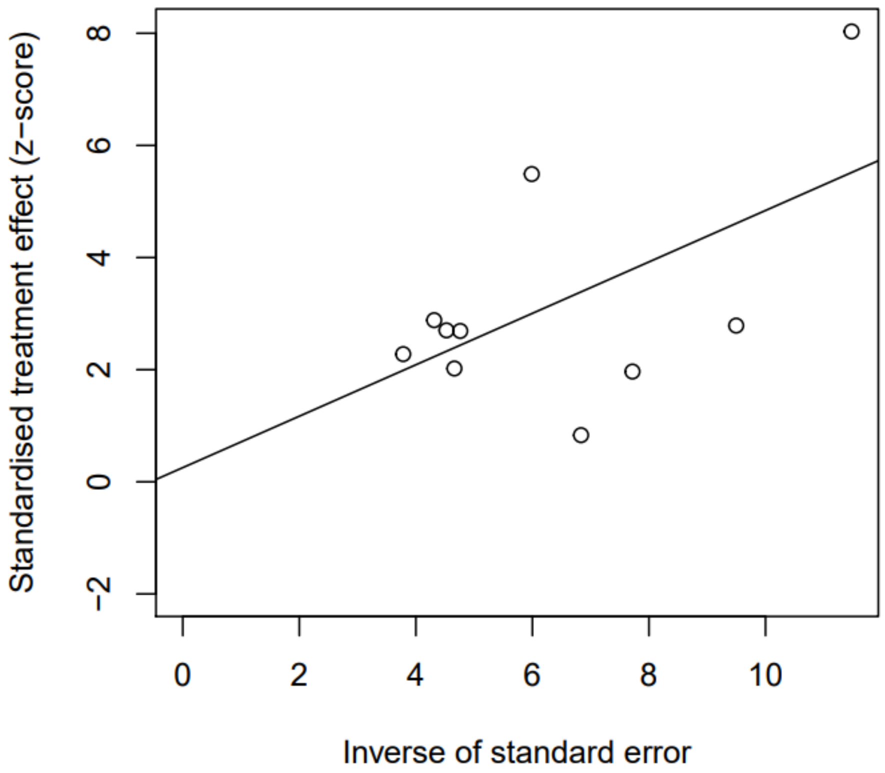

Figure S3. Mechanism underlying the interaction between atopic diseases and ADHD

1. Czarnowicki et al., 2015[1] 2. Czarnowicki et al., 2020[2] 3. Looman et al., 2020[3] 4. Tumes et al., 2017[4] 5. Park et al., 2018[5] 6. Xing et al., 2016[6] 7. Banks 2015[7] 8. Buske-Kirschbaum et al., 2013[8] 9. Kim et al., 2018[9] 10. Levy et al., 2008[10] 11. Puiu et al., 2018[11] 12. Buske-Kirschbaum et al., 2002[12] 13. Chamlin et al., 2004[13] 14. Lin et al., 2017[14] 15. McCrory et al., 2010[15] 16. Nicolaides et al., 2015[16] 17. Quinlan et al., 2017[17] 18. Buske-Kirschbaum et al., 2019[18] 19. Lau et al., 2012[19] 20. Tsai 2006[20] 21. Wamboldt et al., 2000[21] 22. Cowell et al., 2019[22] 23. Van den Bergh et al., 2017[23]

Abbreviation: ADHD, Attention-deficit/hyperactivity disorder; BBB, blood–brain barrier; PFC, prefrontal cortex; ACC, anterior cingulate cortex; HPA axis, hypothalamic–pituitary–adrenal axis; SAM, sympathomedullary pathway

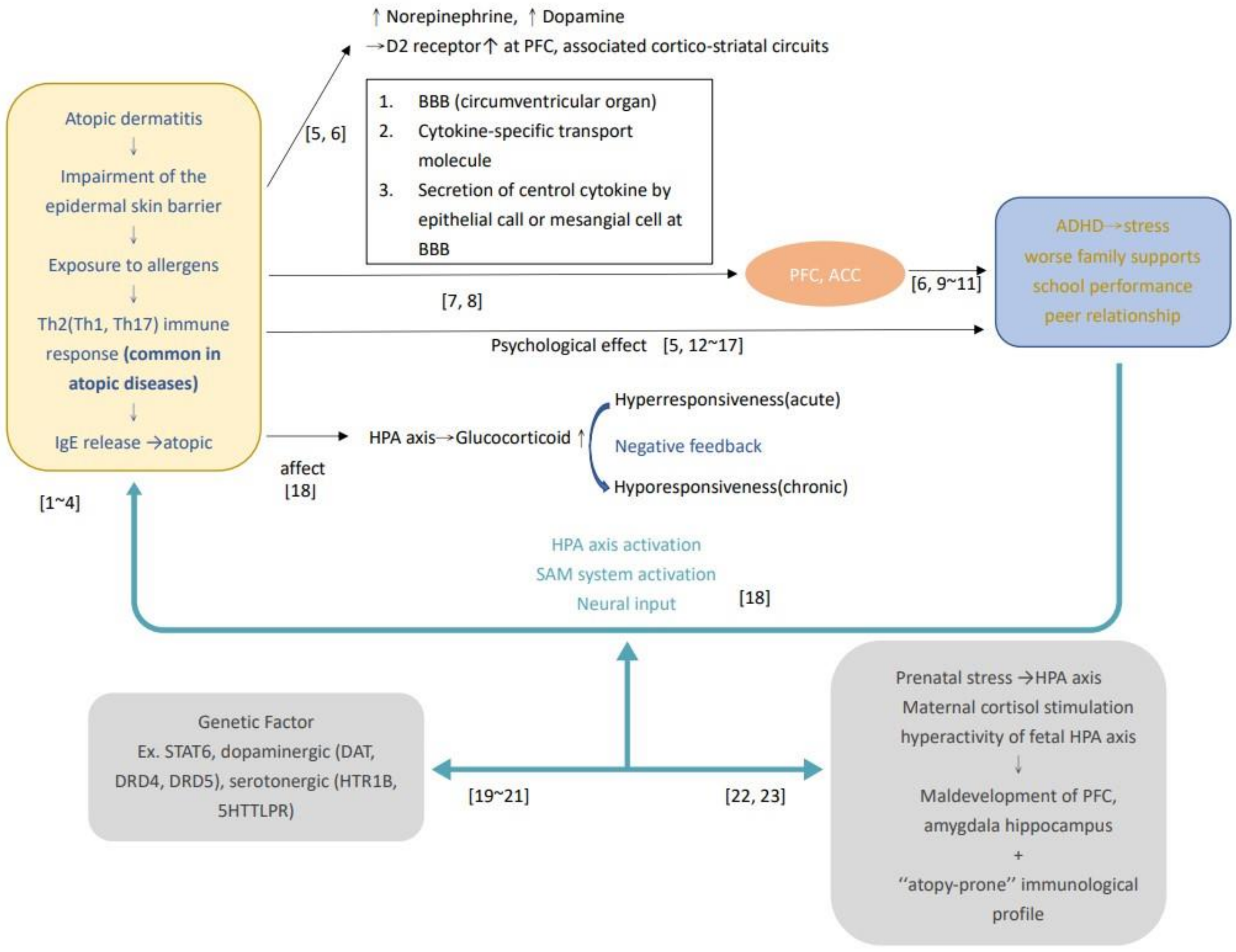

Figure S4. Comparison of immune responses and related effects between acute and chronic phases of atopic diseases

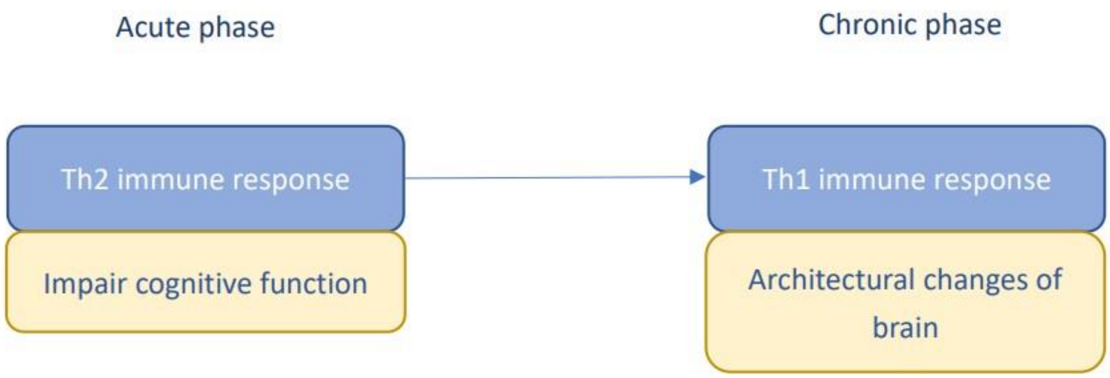

Table

Table S1. Detailed information of studies included in the qualitative analysis

	Study
Outcome type	OR/ Hazard ratios: (Goodwin et al., 2013, Lin et al., 2016, Strom et al., 2016)[24-26]; Fuhrmann et al., 2020[27]; Huang et al., 2020[28]; Jackson-Cowan et al., 2020[29]; Sollander et al., 2020[30]; Wan et al., 2020[31]; Chai et al., 2021[32]; Hou et al., 2021[33]; Vittrup et al., 2021[34]<br>Continuous data for HI, IN, or total ADHD symptoms: (Yuksel et al., 2008, Camfferman et al., 2010, Chang et al., 2013, Kim et al., 2014, Lee et al., 2014, Yang et al., 2014, Catal et al., 2016, Hammer-Helmich et al., 2016, Yang et al., 2016, Feng et al., 2017, Kuniyoshi et al., 2018, Schmitt et al., 2018, L et al., 2019, Feng et al., 2020, Guo et al., 2020, Kuo et al., 2020) [35-50]; Minatoya et al., 2020[51], Montalbano et al., 2020[52]; Berzosa-Grande et al., 2021[53]; Galéra et al., 2021[54]; Özyurt et al., 2021[55]; Rajhans et al., 2021[56]; Yüksel et al., 2021[57]<br>Prevalence/percentage: (Abd El-Hamid et al., 2018, Tajdini et al., 2019, Zhou et al., 2019) [58-60]<br>Other: Keller et al., 2021[61]
Exposed	Atopy in patients with ADHD: (Kim et al., 2014, Abd El-Hamid et al., 2018)[49,58]; Yüksel et al., 2021[57]<br>Pure atopy and healthy controls: (Yuksel et al., 2008, Yang et al., 2014, Schmitt et al., 2018, Feng et al., 2020) [38,42,45,46]<br>Atopy and nonatopic groups: (Camfferman et al., 2010, Chang et al., 2013, Goodwin et al., 2013, Lee et al., 2014, Catal et al., 2016, Hammer-Helmich et al., 2016, Lin et al., 2016, Strom et al., 2016, Kuniyoshi et al., 2018, L et al., 2019, Zhou et al., 2019, Guo et al., 2020, Fuhrmann et al., 2020[27], Kuo et al., 2020) [24-26,35,36,39,40,43,44,47,48,50,60]; Huang et al., 2020[28] ; Jackson-Cowan et al., 2020[29]; Minatoya et al., 2020[51]; Sollander et al., 2020[30]; Wan et al., 2020[31]; Berzosa-Grande et al., 2021[53]; Galéra et al., 2021[54]; Hou et al., 2021[33]<br>Atopy patients and healthy controls: (Yang et al., 2016, Feng et al., 2017, Tajdini et al., 2019)[37,41,59]; Chai et al., 2021[32]; Keller et al., 2021[61]; Özyurt et al., 2021[55]; Rajhans et al., 2021[56]<br>ADHD (symptoms) in patients with atopy: Montalbano et al., 2020[52]; Vittrup et al., 2021[34]
Other variables	**Description for potential confounding factors**<br>Age and sex: (Yang et al., 2014, Yang et al., 2016, Feng et al., 2017, Feng et al., 2020)[37,38,41,42]; Fuhrmann et al., 2020[27]; Jackson-Cowan et al., 2020[29]; Sollander et al., 2020[30]; Minatoya et al., 2020[51]; Montalbano et al., 2020[52]; Wan et al., 2020[31]; Berzosa-Grande et al.,

	2021[53]; Galéra et al., 2021[54]; Hou et al., 2021[33]; Özyurt et al., 2021[55]; Rajhans et al., 2021[56]; Vittrup et al., 2021[34]; Yüksel et al., 2021[57]<br>Birth Hx: Feng et al., 2020[42]; Galéra et al., 2021[54]; Yüksel et al., 2021[57]<br>Parents' stress or mental health: (Goodwin et al., 2013, Lee et al., 2014, Schmitt et al., 2018)[24,36,45]; Kuo et al., 2020[44]; Montalbano et al., 2020[52]; Berzosa-Grande et al., 2021[53]; Galéra et al., 2021[54]<br>Primary caregiver's education: (Goodwin et al., 2013, Feng et al., 2020)[24,42]; Sollander et al., 2020[30]; Wan et al., 2020[31]; Galéra et al., 2021[54]; Hou et al., 2021[33]; Özyurt et al., 2021[55]<br>Family income: Jackson-Cowan et al., 2020[29]; Wan et al., 2020[31]; Galéra et al., 2021[54]; Hou et al., 2021[33]; Vittrup et al., 2021[34]<br>Environment (tobacco): (Goodwin et al., 2013, Feng et al., 2017, Tajdini et al., 2019)[24,41,59]<br>Sleep disturbance: (Camfferman et al., 2010, Lin et al., 2016, Strom et al., 2016, Schmitt et al., 2018, Feng et al., 2020)[25,26,40,42,45]<br>Other physical conditions: Strom et al., 2016[26]; Wan et al., 2020[31]; Galéra et al., 2021[54]; Hou et al., 2021[33]; Vittrup et al., 2021[34]; Yüksel et al., 2021[57]<br>Mental health: Jackson-Cowan et al., 2020[29]; Galéra et al., 2021[54]<br>**Assessment for atopic diseases**<br>Eosinophils in peripheral blood: (Chang et al., 2013)[47]<br>IgE and allergens: (Guo et al., 2020)[48]<br>Symptoms: (Yang et al., 2014, Yang et al., 2016, Feng et al., 2017, Schmitt et al., 2018, Feng et al., 2020)[37,38,41,42,45]; Rajhans et al., 2021[56]<br>Persistent atopic diseases: (Yang et al., 2014, Yang et al., 2016, Feng et al., 2017)[37,38,41]<br>Severity of atopic diseases: (Yang et al., 2014, Yang et al., 2016, Tajdini et al., 2019, Zhou et al., 2019, Feng et al., 2020)[37,38,42,59,60]; Minatoya et al., 2020[51]; Montalbano et al., 2020[52]; Berzosa-Grande et al., 2021[53]<br>Atopic disease onset: (Schmitt et al., 2018, Tajdini et al., 2019, Zhou et al., 2019, Feng et al., 2020)[42,45,59,60]; Fuhrmann et al., 2020[27]; Rajhans et al., 2021[56]<br>Duration: (Zhou et al., 2019, Feng et al., 2020)[42,60]; Rajhans et al., 2021[56]<br>Positive allergens: Feng et al., 2017[41]; Berzosa-Grande et al., 2021[53]<br>Treatments: (Schmitt et al., 2018, Tajdini et al., 2019, Zhou et al., 2019, Feng et al., 2020)[42,45,59,60]<br>Family history of atopic diseases: (Yang et al., 2014, Yang et al., 2016, Tajdini et al., 2019, Zhou et al., 2019)[37,38,59,60]; Minatoya et al., 2020[51]<br>Multiple atopic diseases: (Yang et al., 2014, Yang et al., 2016, Feng et al., 2017, Schmitt et al., 2018, Tajdini et al., 2019)[37,38,41,45,59]; Jackson-Cowan et al., 2020[29]; Sollander et al., 2020[30]; Berzosa-Grande et al., 2021[53]; Vittrup et al., 2021[34]<br>**Other assessment for ADHD**<br>Percentage of abnormal scores: (Hammer-Helmich et al., 2016, Feng et al., 2020)[35,42]

	Quality of life: (Feng et al., 2017, Schmitt et al., 2018, Feng et al., 2020)[41,42,45]<br>CPT/CAT: (Kim et al., 2014, Yang et al., 2014, Yang et al., 2016)[37,38,49]
Subgroup	Treatments: (Chang et al., 2013, Kim et al., 2014, Yang et al., 2016)[37,47,49], Fuhrmann et al., 2020[27]<br>Age: (Strom et al., 2016, Kuniyoshi et al., 2018)[26,43]; Berzosa-Grande et al., 2021[53]; Hou et al., 2021[33]<br>Sex: Jackson-Cowan et al., 2020[29]<br>Severity of atopic diseases: (Chang et al., 2013, Goodwin et al., 2013, Strom et al., 2016, Kuniyoshi et al., 2018)[24,26,43,47]; Montalbano et al., 2020[52]<br>Pure atopy (without ADHD), total atopy, pure ADHD (without atopy): Yang et al., 2014[38]; Galéra et al., 2021[54]<br>Primary caregiver's education: Hammer-Helmich et al., 2016[35], Sollander et al., 2020[30]<br>Multiple atopic diseases: Hammer-Heimich et al., 2016[35]; Kuo et al., 2020[44], Strom et al., 2016[26], Sollander et al., 2020[30]<br>Atopy with or without ADHD symptoms: Feng et al., 2020[42]<br>ADHD type: (Lin et al., 2016, Zhou et al., 2019)[25,60]<br>Comorbidity of ADHD: (Yang et al., 2014, Yang et al., 2016)[37,38]

Abbreviations: OR, odds ratio; ADHD, attention-deficit/hyperactivity disorder; CPT/CAT, (Continuous Performance Test/Comprehensive Attention Test)

Table S2. Detailed information on the risk of bias assessment

Risk of bias assessment

The quality of included studies was determined using the Joanna Briggs Institute's critical appraisal checklist for systematic reviews [62]. We used this checklist to evaluate case–control and cross-sectional study designs. The checklist for cross-sectional studies consisted of 10 questions assessing the following factors: (1) use of clearly defined inclusion criteria, (2) detailed description of study objects and settings, (3) exposure measurement, (4) inclusion of objective and standard criteria for the measurement of the condition (5) strategies adopted to identify confounding factors (questions 5 and 6 were related to this factor), (6) strategies adopted to deal with confounding factors (7) outcome measurement, and (8) use of appropriate statistical analysis methods. Each question had the following response options: yes, no, unclear, and not applicable. The overall appraisal of whether to include, exclude, or seek further information on studies according to the results of questions mentioned above.

Risk of bias

Table mentioned below lists the results of the overall risk of bias[63]. The complete form used to examine the risk of bias is shown below. The studies were all cross-sectional studies. In these studies, the risk of bias was mainly related to not providing a detailed description of study participants, and the settings and strategies adopted to identify and manage confounding factors. One study[44] was determined was given the note "seek further information" because it did not provide detailed information regarding the inclusion of both study participants and settings and strategies adopted to identify and manage confounding factors. None of the studies had an overall appraisal category of "exclusion." Most studies provided detailed information regarding strategies adopted to identify and manage confounding factors.

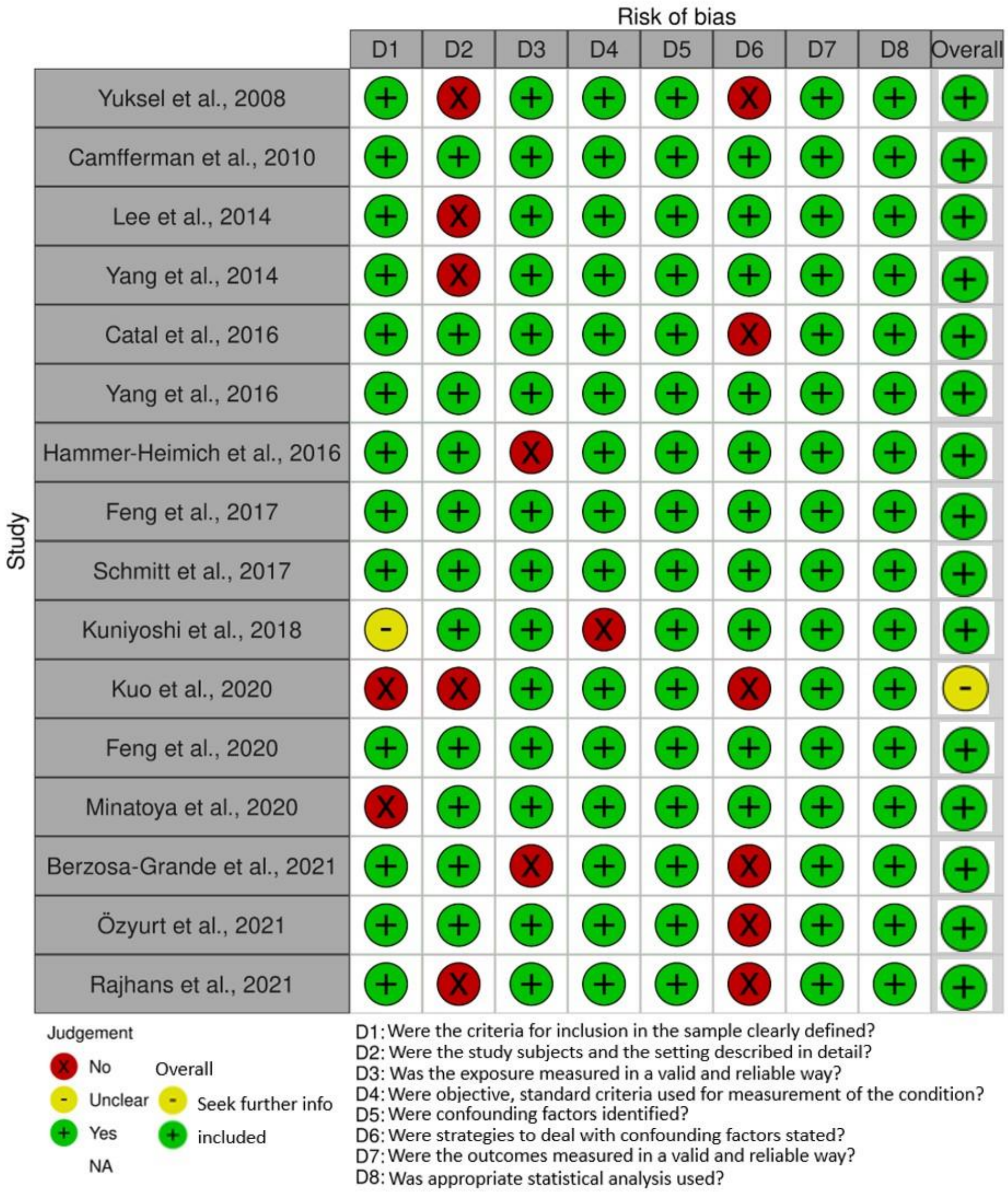
Risk of bias
D1
D2
D3
D4
D5
D6
D7
D8
Overall
Study
Yuksel et al., 2008
Camfferman et al., 2010
Lee et al., 2014
Yang et al., 2014
Catal et al., 2016
Yang et al., 2016
Hammer-Heimich et al., 2016
Feng et al., 2017
Schmitt et al., 2017
Kuniyoshi et al., 2018
Kuo et al., 2020
Feng et al., 2020
Minatoya et al., 2020
Berzosa-Grande et al., 2021
Özyurt et al., 2021
Rajhans et al., 2021
Judgement
No
Unclear
Yes
NA
Overall
Seek further info
included
D1: Were the criteria for inclusion in the sample clearly defined?
D2: Were the study subjects and the setting described in detail?
D3: Was the exposure measured in a valid and reliable way?
D4: Were objective, standard criteria used for measurement of the condition?
D5: Were confounding factors identified?
D6: Were strategies to deal with confounding factors stated?
D7: Were the outcomes measured in a valid and reliable way?
D8: Was appropriate statistical analysis used?

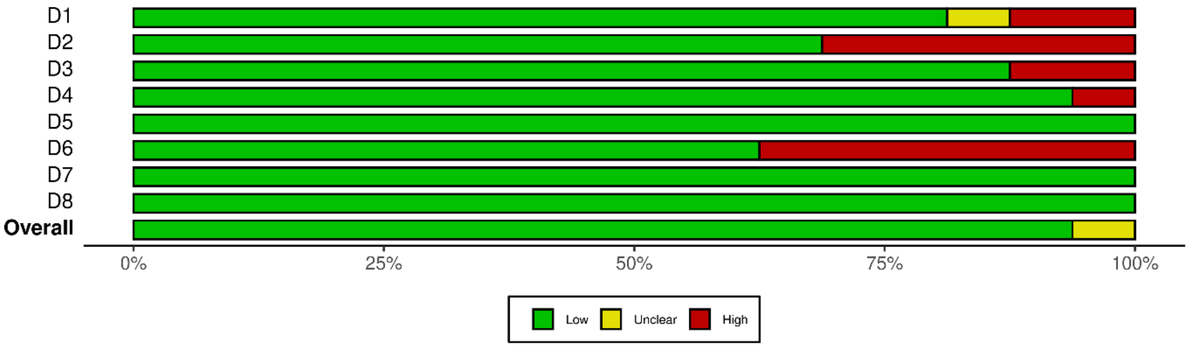
D1
D2
D3
D4
D5
D6
D7
D8
Overall
0%
25%
50%
75%
100%
Low
Unclear
High

Appendix C. References to articles solely included in qualitative synthesis

Abd El-Hamid, Z. B., M. M. Refaat, H. H. El-Shahawy, A. M. Eissa, M. M. El-Sheikh, A. S. Abdel-Rehim, S. A. Elgaaly, M. H. Abd El-Moneam, N. A. Mohamed and O. M. Abdel Latif , 2018. Impact of allergy on children with attention deficit hyperactivity disorder. Eur Ann Allergy Clin Immunol 50(6), 262-267. https://doi.org/10.23822/EurAnnACI.1764-1489.72

Berzosa-Grande, M. P., González-Fraile, E., Sánchez-López, R., Soria-Oliver, M. & Rueda-Esteban, S. The relationship between allergic diseases and internalising and externalising behaviours in Spanish children: A cross-sectional study. Allergol Immunopathol (Madr) 49, 65-72, https://doi.org/10.15586/aei.v49i3.78(2021).

Chai, P. H., Chang, S. & Cawthorpe, D. The temporal hyper-morbidity of asthma and attention deficit disorder: Implications for interpretation based on comparison of prospective and cross-sectional population samples. Psychiatry Investigation 18, 166-171, https://doi.org/10.30773/pi.2020.0349(2021).

Chang, H. Y., J. H. Seo, H. Y. Kim, J. W. Kwon, B. J. Kim, H. B. Kim, S. Y. Lee, G. C. Jang, D. J. Song, W. K. Kim, J. Y. Shim, H. J. Kim, J. W. Park, S. H. Cho, J. S. Lee, Y. J. Shin and S. J. Hong,2013. Allergic diseases in preschoolers are associated with psychological and behavioural problems. Allergy Asthma Immunol Res 5(5), 315-321. https://doi.org/10.4168/aair.2013.5.5.315

Fuhrmann, S., Tesch, F., Romanos, M., Abraham, S. & Schmitt, J. ADHD in school-age children is related to infant exposure to systemic H1-antihistamines. *Allergy: European Journal of Allergy and Clinical Immunology* **75**, 2956-2957, https://doi.org/10.1111/all.14411 (2020).

Galéra, C. et al. Medical conditions and Attention-Deficit/Hyperactivity Disorder symptoms from early childhood to adolescence. Molecular Psychiatry, https://doi.org/10.1038/s41380-021-01357-x(2021)

Goodwin, R. D., M. Robinson, P. D. Sly, I. W. McKeague, E. S. Susser, S. R. Zubrick, F. J. Stanley and E. Mattes,2013. Severity and persistence of asthma and mental health: a birth cohort study. Psychol Med 43(6), 1313-1322. https://doi.org/10.1017/S0033291712001754

Guo, M. M. H., L. J. Wang, T. Y. Hsu, K. D. Yang and H. C. Kuo, 2020. Peanut sensitivity and allergic rhinitis in young children are associated with attention-deficit hyperactivity disorder symptoms in adolescence. Neuropsychiatric Disease and Treatment 16, 1349-1357. https://doi.org/10.2147/NDT.S232299

Hou, A. & Silverberg, J. I. Predictors and age-dependent pattern of psychologic problems in childhood atopic dermatitis. Pediatric Dermatology 38, 606-612, https://doi.org/10.1111/pde.14588(2021).

Huang, A. H., Y. S. Roh, N. Sutaria, J. Choi, K. A. Williams, J. K. Canner, A. L. Grossberg and S. G. Kwatra,2021. Real-world comorbidities of atopic dermatitis in the pediatric ambulatory population in the United States. J Am Acad Dermatol. https://doi.org/10.1016/j.jaad.2021.03.016

Jackson-Cowan, L., E. F. Cole, J. I. Silverberg and L. P. Lawley,2020. Childhood atopic dermatitis is associated with cognitive dysfunction: A National Health Interview Survey study from 2008 to 2018. Ann Allergy Asthma Immunol. https://doi.org/10.1016/j.anai.2020.11.008

Keller, W. et al. Atopic diseases in children and adolescents are associated with behavioural difficulties. BMC Pediatrics 21, https://doi.org/10.1186/s12887-021-02663-7(2021).

L, L. K., A. Cices, A. B. Fishbein and A. S. Paller,2019. Neurocognitive function in moderate-severe pediatric atopic dermatitis: A case-control study. Pediatr Dermatol 36(1), 110-114. https://doi.org/10.1111/pde.13710

Minatoya, M., Suyama, S. & Kishi, R. [Relationship between atopic dermatitis and children's mental and behavioral health: The Hokkaido Study]. *Nihon Koshu Eisei Zasshi* **67**, 745-751, https://doi.org/10.11236/jph.67.10_745(2020).

Montalbano, L. *et al.* Relationship between quality of life and behavioural disorders in children with persistent asthma: a Multiple Indicators Multiple Causes (MIMIC) model. *Sci Rep* **10**, 6957, https://doi.org/10.1038/s41598-020-62264-9(2020).

Özyurt, G. et al. Adolescents with asthma reported more peer victimization, more anger repression, and less anger expression. J Asthma 58, 1307-1313, https://doi.org/10.1080/02770903.2020.1782428(2021)

Rajhans, P., Sagar, R., Patra, B. N., Bhargava, R. & Kabra, S. K. Psychiatric Morbidity and Behavioral Problems in Children and Adolescents with Bronchial Asthma. Indian Journal of Pediatrics 88, 968-973, https://doi.org/10.1007/s12098-021-03661-4(2021)

Edvinsson Sollander, S., H. Fabian, A. Sarkadi, R. Salari, E. Fält, A. Dahlberg, I. Feldman and N. Durbeej,2020. Asthma and allergies correlate with mental health problems in preschool children. Acta Paediatr. https://doi.org/10.1111/apa.15709

Strom, M. A., A. B. Fishbein, A. S. Paller and J. I. Silverberg,2016. Association between atopic dermatitis and attention deficit hyperactivity disorder in U.S. children and adults. Br J Dermatol 175(5), 920-929. https://doi.org/10.1111/bjd.14697

Vittrup, I. *et al.* Association between hospital-diagnosed atopic dermatitis and psychiatric disorders and medication use in childhood. *Br J Dermatol* **185**, 91-100, https://doi.org/10.1111/bjd.19817(2021)

Wan, J., Takeshita, J., Shin, D. B. & Gelfand, J. M. Mental health impairment among children with atopic dermatitis: A United States population-based cross-sectional study of the 2013-

2017 National Health Interview Survey. *Journal of the American Academy of Dermatology* **82**, 1368-1375, https://doi.org/10.1016/j.jaad.2019.10.019(2020).
Yüksel, A. E. et al. Adhd and its associations with pregnancy, birth, developmental and medical-related characteristics. Current Psychology: A Journal for Diverse Perspectives on Diverse Psychological Issues, https://doi.org/10.1007/s12144-021-01817-1(2021)
Zhou, H., Z. Chen, W. Zhao, Y. Liu and Y. Cui, 2019. Evaluation of neuropsychiatric comorbidities and their clinical characteristics in Chinese children with asthma using the MINI kid tool. BMC Pediatr 19(1), 454. https://doi.org/10.1186/s12887-019-1834-7

Appendix D. References to articles solely included in meta-analysis

Catal, F., E. Topal, N. Soylu, O. Ozel Ozcan, M. H. Celiksoy, A. Babayiğit, H. T. Karakoç, D. Erge and R. Sancak, 2016. Psychiatric disorders and symptoms severity in preschool children with atopic eczema. Allergol Immunopathol (Madr) 44(2), 120-124 https://doi.org/.10.1016/j.aller.2015.04.006

Berzosa-Grande, M. P., González-Fraile, E., Sánchez-López, R., Soria-Oliver, M. & Rueda-Esteban, S. The relationship between allergic diseases and internalising and externalising behaviours in Spanish children: A cross-sectional study. Allergol Immunopathol (Madr) 49, 65-72, https://doi.org/10.15586/aei.v49i3.78(2021).

Feng, B., H. Jin, H. Xiang, B. Li, X. Zheng, R. Chen, Y. Shi, S. Chen and B. Chen, 2017. Association of pediatric allergic rhinitis with the ratings of attention-deficit/hyperactivity disorder. Am J Rhinol Allergy 31(3), 161-167. https://doi.org/10.2500/ajra.2017.31.4439

Feng, L. J., A. W. Chen, X. Y. Luo and H. Wang,2020. Increased attention deficit/hyperactivity and oppositional defiance symptoms of 6-12 years old Chinese children with atopic dermatitis. Medicine (Baltimore) 99(25), e20801. https://doi.org/10.1097/MD.0000000000020801

Lee, Y. S., S. H. Kim, J. H. You, H. T. Baek, C. Na, B. N. Kim and D. H. Han,2014. Attention deficit hyperactivity disorder like behavioral problems and parenting stress in pediatric allergic rhinitis. Psychiatry Investig 11(3), 266-271. https://doi.org/10.4306/pi.2014.11.3.266

Minatoya, M., Suyama, S. & Kishi, R. [Relationship between atopic dermatitis and children's mental and behavioral health: The Hokkaido Study]. *Nihon Koshu Eisei Zasshi* **67**, 745-751, https://doi.org/10.11236/jph.67.10_745(2020).

Özyurt, G. et al. Adolescents with asthma reported more peer victimization, more anger repression, and less anger expression. J Asthma 58, 1307-1313, https://doi.org/10.1080/02770903.2020.1782428(2021)

Rajhans, P., Sagar, R., Patra, B. N., Bhargava, R. & Kabra, S. K. Psychiatric Morbidity and Behavioral Problems in Children and Adolescents with Bronchial Asthma. Indian Journal of Pediatrics 88, 968-973, https://doi.org/10.1007/s12098-021-03661-4(2021)

Yang, M. T., W. T. Lee, J. S. Liang, Y. J. Lin, W. M. Fu and C. C. Chen, 2014. Hyperactivity and impulsivity in children with untreated allergic rhinitis: Corroborated by rating scale and continuous performance test. Pediatrics and Neonatology 55(3), 168-174. https://doi.org/10.1016/j.pedneo.2013.09.003

Yuksel, H., A. Sogut and O. Yilmaz, 2008. Attention deficit and hyperactivity symptoms in

children with asthma. J Asthma 45(7), 545-547. https://doi.org/10.1080/02770900801990016

Appendix E. Reference to articles included in supplementary materials

1 Czarnowicki, T. *et al.* Early pediatric atopic dermatitis shows only a cutaneous lymphocyte antigen (CLA)(+) TH2/TH1 cell imbalance, whereas adults acquire CLA(+) TH22/TC22 cell subsets. *J Allergy Clin Immunol* **136**, 941-951.e943, doi:10.1016/j.jaci.2015.05.049 (2015).

2 Czarnowicki, T. *et al.* Evolution of pathologic T-cell subsets in patients with atopic dermatitis from infancy to adulthood. *J Allergy Clin Immunol* **145**, 215-228, doi:10.1016/j.jaci.2019.09.031 (2020).

3 Looman, K. I. M. *et al.* Associations of Th2, Th17, Treg cells, and IgA(+) memory B cells with atopic disease in children: The Generation R Study. *Allergy* **75**, 178-187, doi:10.1111/all.14010 (2020).

4 Tumes, D. J. *et al.* Epigenetic regulation of T-helper cell differentiation, memory, and plasticity in allergic asthma. *Immunol Rev* **278**, 8-19, doi:10.1111/imr.12560 (2017).

5 Park, G., Jung, Y. S., Park, M. K., Yang, C. H. & Kim, Y. U. Melatonin inhibits attention-deficit/hyperactivity disorder caused by atopic dermatitis-induced psychological stress in an NC/Nga atopic-like mouse model. *Sci Rep* **8**, 14981, doi:10.1038/s41598-018-33317-x (2018).

6 Xing, B., Li, Y. C. & Gao, W. J. Norepinephrine versus dopamine and their interaction in modulating synaptic function in the prefrontal cortex. *Brain Res* **1641**, 217-233, doi:10.1016/j.brainres.2016.01.005 (2016).

7 Banks, W. A. The blood-brain barrier in neuroimmunology: Tales of separation and assimilation. *Brain Behav Immun* **44**, 1-8, doi:10.1016/j.bbi.2014.08.007 (2015).

8 Buske-Kirschbaum, A. *et al.* Psychoendocrine and psychoneuroimmunological mechanisms in the comorbidity of atopic eczema and attention deficit/hyperactivity disorder. *Psychoneuroendocrinology* **38**, 12-23, doi:10.1016/j.psyneuen.2012.09.017 (2013).

9 Kim, J. I. *et al.* Interaction between DRD2 and lead exposure on the cortical thickness of the frontal lobe in youth with attention-deficit/hyperactivity disorder. *Prog Neuropsychopharmacol Biol Psychiatry* **82**, 169-176, doi:10.1016/j.pnpbp.2017.11.018 (2018).

10 Levy, F. Pharmacological and therapeutic directions in ADHD: Specificity in the PFC. *Behav Brain Funct* **4**, 12, doi:10.1186/1744-9081-4-12 (2008).

11 Puiu, A. A. *et al.* Impulsive aggression and response inhibition in attention-deficit/hyperactivity disorder and disruptive behavioral disorders: Findings from a systematic review. *Neurosci Biobehav Rev* **90**, 231-246, doi:10.1016/j.neubiorev.2018.04.016 (2018).

12 Buske-Kirschbaum, A., Gierens, A., Höllig, H. & Hellhammer, D. H. Stress-induced immunomodulation is altered in patients with atopic dermatitis. *J Neuroimmunol* **129**, 161-167, doi:10.1016/s0165-5728(02)00168-6 (2002).

13 Chamlin, S. L., Frieden, I. J., Williams, M. L. & Chren, M. M. Effects of atopic dermatitis on young American children and their families. *Pediatrics* **114**, 607-611, doi:10.1542/peds.2004-0374 (2004).

14 Lin, T. K., Zhong, L. & Santiago, J. L. Association between Stress and the HPA Axis in the Atopic Dermatitis. *Int J Mol Sci* **18**, doi:10.3390/ijms18102131 (2017).

15 McCrory, E., De Brito, S. A. & Viding, E. Research review: the neurobiology and genetics of maltreatment and adversity. *J Child Psychol Psychiatry* **51**, 1079-1095, doi:10.1111/j.1469-7610.2010.02271.x (2010).

16 Nicolaides, N. C., Kyratzi, E., Lamprokostopoulou, A., Chrousos, G. P. & Charmandari, E. Stress, the stress system and the role of glucocorticoids. *Neuroimmunomodulation* **22**, 6-19, doi:10.1159/000362736 (2015).

17 Quinlan, E. B. *et al.* Psychosocial Stress and Brain Function in Adolescent Psychopathology. *Am J Psychiatry* **174**, 785-794, doi:10.1176/appi.ajp.2017.16040464 (2017).

18 Buske-Kirschbaum, A. *et al.* Altered hypothalamus-pituitary-adrenal axis function: A relevant factor in the comorbidity of atopic eczema and attention deficit/hyperactivity disorder? *Psychoneuroendocrinology* **105**, 178-186, doi:10.1016/j.psyneuen.2018.12.005 (2019).

19 Lau, M. *et al.* Loss of STAT6 promotes autoimmune disease and atopy on a susceptible genetic background. *J Autoimmun* **39**, 388-397, doi:10.1016/j.jaut.2012.06.003 (2012).

20 Tsai, S. J. Signal transducer and activator of transcription 6 (STAT6) and attention-deficit hyperactivity disorder: a speculative hypothesis. *Med Hypotheses* **67**, 1342-1344, doi:10.1016/j.mehy.2006.05.019 (2006).

21 Wamboldt, M. Z. *et al.* Familial association between allergic disorders and depression in adult Finnish twins. *Am J Med Genet* **96**, 146-153, doi:10.1002/(sici)1096-8628(20000403)96:2<146::aid-ajmg4>3.0.co;2-j (2000).

22 Cowell, W. J., Bellinger, D. C., Wright, R. O. & Wright, R. J. Antenatal active maternal asthma and other atopic disorders is associated with ADHD behaviors among school-aged children. *Brain Behav Immun* **80**, 871-878, doi:10.1016/j.bbi.2019.05.040 (2019).

23 Van den Bergh, B. R. H. *et al.* Prenatal developmental origins of behavior and mental health: The influence of maternal stress in pregnancy. *Neurosci Biobehav Rev* **117**, 26-64, doi:10.1016/j.neubiorev.2017.07.003 (2020).
24 Goodwin, R. D. *et al.* Severity and persistence of asthma and mental health: a birth cohort study. *Psychol Med* **43**, 1313-1322, doi:10.1017/s0033291712001754 (2013).
25 Lin, Y. T. *et al.* Associations between allergic diseases and attention deficit hyperactivity/oppositional defiant disorders in children. *Pediatric Research* **80**, 480-485, doi:10.1038/pr.2016.111 (2016).
26 Strom, M. A., Fishbein, A. B., Paller, A. S. & Silverberg, J. I. Association between atopic dermatitis and attention deficit hyperactivity disorder in U.S. children and adults. *Br J Dermatol* **175**, 920-929, doi:10.1111/bjd.14697 (2016).
27 Fuhrmann, S., Tesch, F., Romanos, M., Abraham, S. & Schmitt, J. ADHD in school-age children is related to infant exposure to systemic H1-antihistamines. *Allergy: European Journal of Allergy and Clinical Immunology* **75**, 2956-2957, doi:10.1111/all.14411 (2020).
28 Huang, A. H. *et al.* Real-world comorbidities of atopic dermatitis in the pediatric ambulatory population in the United States. *J Am Acad Dermatol*, doi:10.1016/j.jaad.2021.03.016 (2021).
29 Jackson-Cowan, L., Cole, E. F., Silverberg, J. I. & Lawley, L. P. Childhood atopic dermatitis is associated with cognitive dysfunction: A National Health Interview Survey study from 2008 to 2018. *Ann Allergy Asthma Immunol* **126**, 661-665, doi:10.1016/j.anai.2020.11.008 (2021).
30 Edvinsson Sollander, S. *et al.* Asthma and allergies correlate with mental health problems in preschool children. *Acta Paediatr* **110**, 1601-1609, doi:10.1111/apa.15709 (2021).
31 Wan, J., Takeshita, J., Shin, D. B. & Gelfand, J. M. Mental health impairment among children with atopic dermatitis: A United States population-based cross-sectional study of the 2013-2017 National Health Interview Survey. *Journal of the American Academy of Dermatology* **82**, 1368-1375, doi:10.1016/j.jaad.2019.10.019 (2020).
32 Chai, P. H., Chang, S. & Cawthorpe, D. The temporal hyper-morbidity of asthma and attention deficit disorder: Implications for interpretation based on comparison of prospective and cross-sectional population samples. *Psychiatry Investigation* **18**, 166-171, doi:10.30773/pi.2020.0349 (2021).
33 Hou, A. & Silverberg, J. I. Predictors and age-dependent pattern of psychologic problems in childhood atopic dermatitis. *Pediatric Dermatology* **38**, 606-612, doi:10.1111/pde.14588 (2021).
34 Vittrup, I. *et al.* Association between hospital-diagnosed atopic dermatitis and psychiatric disorders and medication use in childhood. *Br J Dermatol* **185**, 91-100,

doi:10.1111/bjd.19817 (2021).
35 Hammer-Helmich, L. *et al.* Mental health associations with eczema, asthma and hay fever in children: a cross-sectional survey. *BMJ Open* **6**, e012637, doi:10.1136/bmjopen-2016-012637 (2016).
36 Lee, Y. S. *et al.* Attention deficit hyperactivity disorder like behavioral problems and parenting stress in pediatric allergic rhinitis. *Psychiatry Investig* **11**, 266-271, doi:10.4306/pi.2014.11.3.266 (2014).
37 Yang, M. T. *et al.* Attention-deficit/hyperactivity disorder-related symptoms improved with allergic rhinitis treatment in children. *American Journal of Rhinology and Allergy* **30**, 209-214, doi:10.2500/ajra.2016.30.4301 (2016).
38 Yang, M. T. *et al.* Hyperactivity and impulsivity in children with untreated allergic rhinitis: Corroborated by rating scale and continuous performance test. *Pediatrics and Neonatology* **55**, 168-174, doi:10.1016/j.pedneo.2013.09.003 (2014).
39 Catal, F. *et al.* Psychiatric disorders and symptoms severity in preschool children with atopic eczema. *Allergologia et Immunopathologia* **44**, 120-124, doi:10.1016/j.aller.2015.04.006 (2016).
40 Camfferman, D. *et al.* Eczema, sleep, and behavior in children. *J Clin Sleep Med* **6**, 581-588 (2010).
41 Feng, B. *et al.* Association of pediatric allergic rhinitis with the ratings of attention-deficit/hyperactivity disorder. *Am J Rhinol Allergy* **31**, 161-167, doi:10.2500/ajra.2017.31.4439 (2017).
42 Feng, L. J., Chen, A. W., Luo, X. Y. & Wang, H. Increased attention deficit/hyperactivity and oppositional defiance symptoms of 6-12 years old Chinese children with atopic dermatitis. *Medicine (Baltimore)* **99**, e20801, doi:10.1097/MD.0000000000020801 (2020).
43 Kuniyoshi, Y. *et al.* Severity of eczema and mental health problems in Japanese schoolchildren: The ToMMo Child Health Study. *Allergol Int* **67**, 481-486, doi:10.1016/j.alit.2018.02.009 (2018).
44 Kuo, H. C., Chang, L. S., Tsai, Z. Y. & Wang, L. J. Allergic diseases do not impair the cognitive development of children but do damage the mental health of their caregivers. *Sci Rep* **10**, 13854, doi:10.1038/s41598-020-70825-1 (2020).
45 Schmitt, J. *et al.* Increased attention-deficit/hyperactivity symptoms in atopic dermatitis are associated with history of antihistamine use. *Allergy: European Journal of Allergy and Clinical Immunology* **73**, 615-626, doi:10.1111/all.13326 (2018).
46 Yuksel, H., Sogut, A. & Yilmaz, O. Attention deficit and hyperactivity symptoms in children with asthma. *Journal of Asthma* **45**, 545-547, doi:10.1080/02770900801990016 (2008).
47 Chang, H. Y. *et al.* Allergic diseases in preschoolers are associated with psychological

and behavioural problems. *Allergy Asthma Immunol Res* **5**, 315-321, doi:10.4168/aair.2013.5.5.315 (2013).
48 Guo, M. M., Wang, L. J., Hsu, T. Y., Yang, K. D. & Kuo, H. C. Peanut Sensitivity and Allergic Rhinitis in Young Children are Associated with Attention-Deficit Hyperactivity Disorder Symptoms in Adolescence. *Neuropsychiatr Dis Treat* **16**, 1349-1357, doi:10.2147/NDT.S232299 (2020).
49 Kim, D. K. *et al.* Treatment of allergic rhinitis is associated with improved attention performance in children: the Allergic Rhinitis Cohort Study for Kids (ARCO-Kids). *PLoS One* **9**, e109145, doi:10.1371/journal.pone.0109145 (2014).
50 L, L. K., Cices, A., Fishbein, A. B. & Paller, A. S. Neurocognitive function in moderate-severe pediatric atopic dermatitis: A case-control study. *Pediatr Dermatol* **36**, 110-114, doi:10.1111/pde.13710 (2019).
51 Minatoya, M., Suyama, S. & Kishi, R. [Relationship between atopic dermatitis and children's mental and behavioral health: The Hokkaido Study]. *Nihon Koshu Eisei Zasshi* **67**, 745-751, doi:10.11236/jph.67.10_745 (2020).
52 Montalbano, L. *et al.* Relationship between quality of life and behavioural disorders in children with persistent asthma: a Multiple Indicators Multiple Causes (MIMIC) model. *Sci Rep* **10**, 6957, doi:10.1038/s41598-020-62264-9 (2020).
53 Berzosa-Grande, M. P., González-Fraile, E., Sánchez-López, R., Soria-Oliver, M. & Rueda-Esteban, S. The relationship between allergic diseases and internalising and externalising behaviours in Spanish children: A cross-sectional study. *Allergol Immunopathol (Madr)* **49**, 65-72, doi:10.15586/aei.v49i3.78 (2021).
54 Galéra, C. *et al.* Medical conditions and Attention-Deficit/Hyperactivity Disorder symptoms from early childhood to adolescence. *Molecular Psychiatry*, doi:10.1038/s41380-021-01357-x (2021).
55 Özyurt, G. *et al.* Adolescents with asthma reported more peer victimization, more anger repression, and less anger expression. *J Asthma* **58**, 1307-1313, doi:10.1080/02770903.2020.1782428 (2021).
56 Rajhans, P., Sagar, R., Patra, B. N., Bhargava, R. & Kabra, S. K. Psychiatric Morbidity and Behavioral Problems in Children and Adolescents with Bronchial Asthma. *Indian Journal of Pediatrics* **88**, 968-973, doi:10.1007/s12098-021-03661-4 (2021).
57 Yüksel, A. E. *et al.* Adhd and its associations with pregnancy, birth, developmental and medical-related characteristics. *Current Psychology: A Journal for Diverse Perspectives on Diverse Psychological Issues*, doi:10.1007/s12144-021-01817-1 (2021).
58 Abd El-Hamid, Z. B. *et al.* Impact of allergy on children with attention deficit hyperactivity disorder. *Eur Ann Allergy Clin Immunol* **50**, 262-267, doi:10.23822/EurAnnACI.1764-1489.72 (2018).

59 Tajdini, M. *et al.* Associations of Behavioral Disorders with Asthma in Iranian Children. *Iran J Allergy Asthma Immunol* **18**, 340-345, doi:10.18502/ijaai.v18i3.1127 (2019).
60 Zhou, H., Chen, Z., Zhao, W., Liu, Y. & Cui, Y. Evaluation of neuropsychiatric comorbidities and their clinical characteristics in Chinese children with asthma using the MINI kid tool. *BMC Pediatr* **19**, 454, doi:10.1186/s12887-019-1834-7 (2019).
61 Keller, W. *et al.* Atopic diseases in children and adolescents are associated with behavioural difficulties. *BMC Pediatrics* **21**, doi:10.1186/s12887-021-02663-7 (2021).
62 Moola S, M. Z., Tufanaru C, Aromataris E, Sears K, Sfetcu R, Currie M, Qureshi R, Mattis P, Lisy K, Mu P-F in *JBI Manual for Evidence Synthesis* (ed Munn Z Aromataris E) (JBI, 2020).
63 McGuinness, L. A. & Higgins, J. P. T. Risk-of-bias VISualization (robvis): An R package and Shiny web app for visualizing risk-of-bias assessments. *Research Synthesis Methods* **n/a**, doi:10.1002/jrsm.1411 (2020).
